# Immune-Related Adverse Events Associated with Anti-PD-1/PD-L1 Treatment for Malignancies: A Meta-Analysis

**DOI:** 10.3389/fphar.2017.00730

**Published:** 2017-10-18

**Authors:** Peng-Fei Wang, Yang Chen, Si-Ying Song, Ting-Jian Wang, Wen-Jun Ji, Shou-Wei Li, Ning Liu, Chang-Xiang Yan

**Affiliations:** ^1^Department of Neurosurgery, Sanbo Brain Hospital, Capital Medical University, Beijing, China; ^2^State Key Laboratory of Cardiovascular Disease, Department of Cardiology, Fuwai Hospital, National Center for Cardiovascular Diseases, Chinese Academy of Medical Sciences and Peking Union Medical College, Beijing, China; ^3^Department of Clinical Medicine, Capital Medical University, Beijing, China; ^4^Key Laboratory, Department of Neurosurgery, The Second Hospital of Yulin, Xi'an Jiaotong University, Xi'an, China

**Keywords:** Anti-PD-1 antibodies, immune-related adverse events, immunotherapy, oncology, nivolumab

## Abstract

**Background:** Treatment of cancers with programmed cell death protein 1 (PD-1) pathway inhibitors can lead to immune-related adverse events (irAEs), which could be serious and even fetal. Therefore, clinicians should be aware of the characteristics of irAEs associated with the use of such drugs.

**Methods:** The MEDLINE, EMBASE, and Cochrane databases were searched to find potential studies using the following strategies: anti-PD-1/PD-L1 treatment; irAEs; and cancer. R© package Meta was used to pool incidence.

**Results:** Forty-six studies representing 12,808 oncologic patients treated with anti-PD-1/PD-L1 agents were included in the meta-analysis. The anti-PD-1/PD-L1 agents included nivolumab, pembrolizumab, atezolizumab, durvalumab, avelumab, and BMS-936559. The tumor types were melanomas, Hodgkin lymphomas, urothelial carcinomas, breast cancers, non-small cell lung cancers, renal cell carcinomas (RCC), colorectal cancers, and others. We described irAEs according to organ systems, namely, the skin (pruritus, rash, maculopapular rash, vitiligo, and dermatitis), endocrine system (hypothyroidism, hyperthyroidism, hypophysitis, thyroiditis, and adrenal insufficiency), digestive system (colitis, diarrhea, pancreatitis, and increased AST/ALT/bilirubin), respiratory system (pneumonitis, lung infiltration, and interstitial lung disease), and urinary system (increased creatinine, nephritis, and renal failure). In patients treated with the PD-1 signaling inhibitors, the overall incidence of irAEs was 26.82% (95% CI, 21.73–32.61; I^2^, 92.80) in any grade and 6.10% (95% CI, 4.85–7.64; I^2^, 52.00) in severe grade, respectively. The development of irAEs was unrelated to the dose of anti-PD-1/PD-L1 agents. The incidence of particular irAEs varied when different cancers were treated with different drugs. The incidence of death due to irAEs was around 0.17%.

**Conclusion:** The occurrence of irAEs was organ-specific and related to drug and tumor types.

## Introduction

Passive immunotherapy for cancer, which involves the transfer of tumor-targeted mono-antibodies and donor T cells, has demonstrated clinical benefits against a variety of solid and hematological malignancies. In contrast to passive immunity techniques, active immunotherapy strategies aim to augment self/anti-tumor responses (Mellman et al., 2011; Callahan et al., 2016; Michot et al., 2016). However, cancer cells always evolve to exploit multiple pathways to resist immune attack. These immunosuppressive pathways are referred to as “immune checkpoints,” which terminate immune responses in the normal physiological state (Pardoll, 2012). Notably, blockade of these immune checkpoints is frequently reported to be superior to traditional treatments in improving survival of oncologic patients (Borghaei et al., 2015; Ribas et al., 2015; Fehrenbacher et al., 2016; Rittmeyer et al., 2017).

Cytotoxic T lymphocyte-associated antigen 4 (CTLA-4) is expressed exclusively in T cells, where it inhibits their activation (Leach et al., 1996). Antagonistic anti-CTLA-4 antibodies, such as ipilimumab and tremelimumab with proven clinical benefits, have been developed (Pardoll, 2012; Callahan et al., 2016). Ipilimumab has been approved by the FDA (Food and Drug Administration) for the treatment of advanced or recurrent melanomas. Programmed cell death protein 1 (PD-1) and its ligand PD-L1 are immune check points that physiologically limit autoimmunity during inflammatory responses (Leach et al., 1996). Monoclonal antibodies against PD-1 or PD-L1, such as nivolumab, pembrolizumab, atezolizumab, avelumab, and durvalumab, have been produced. These PD-1/PD-L1 inhibitors could result in a stable regression of malignancy (Borghaei et al., 2015; Brahmer et al., 2015; Motzer et al., 2015; Ribas et al., 2015; Weber et al., 2015; Fehrenbacher et al., 2016; Herbst et al., 2016; Rittmeyer et al., 2017). Currently, the FDA has approved the use of nivolumab for advanced melanoma, renal cell carcinoma (RCC), and non-small cell lung cancer (NSCLC), and pembrolizumab for advanced melanoma and NSCLC (Costa et al., 2017). Furthermore, combined blockade of CTLA-4 and PD-1/PD-L1 appears to achieve additional clinical benefits (Callahan et al., 2016).

Treatment with immune-checkpoint inhibitors could cause immune-related adverse events (irAEs) by unbalancing the immune system (Chen et al., 2015; Marrone et al., 2016; Kumar et al., 2017). The definition of irAEs is different from that of AEs and treatment-related AEs. AEs that occur potentially due to immunological effects were defined as irAEs. irAEs could be serious, requiring suspension of immunotherapy and possibly leading to death (Bertrand et al., 2015; Chen et al., 2015; Eigentler et al., 2016). Previous studies indicated that the occurrence of irAEs induced by anti PD-1/PD-L1 agents is related to tumor types and organs and is dose-independent (Michot et al., 2016). Additionally, there are reports of delayed occurrence of irAEs after treatment with anti PD-1/PD-L1 agents (Nishino et al., 2015). Therefore, there is a need to understand the characteristics of irAEs associated with anti PD-1/PD-L1 treatments to help us manage them appropriately.

In this study, we present a systematic review and meta-analysis and aim to assess the incidence and characteristics of irAEs in malignancies treated with anti-PD-1/PD-L1 agents.

## Methods

The protocol of this meta-analysis was registered at PROSPERO, International Prospective Register of Systematic Reviews (crd.york.ac.uk/prospero, Identifier: 42016051745).

### Literature searches

MEDLINE, EMBASE, and Cochrane databases were searched to determine potentially eligible studies from database inception to March 1, 2017. We put no restriction on language. The following search terms were used: “safety OR security OR side effects OR adverse events AND (anti-PD-1 OR anti-PD-L1 OR nivolumab OR pembrolizumab OR BMS-936559 OR atezolizumab OR avelumab OR durvalumab)” in MEDLINE and EMBASE, and “anti-PD-1 OR anti-PD-L1 OR nivolumab OR pembrolizumab OR BMS-936559 OR atezolizumab OR avelumab OR durvalumab” in Cochrane databases. We also screened the references of included studies and relevant reviews to find potential studies. There were too many medical records reporting the side effects of anti-PD-1/PD-L1 agents, and it was difficult to check all conference abstracts in the 2 years prior to this study.

### Selection criteria

Eligible studies were required to meet the following criteria: (1) Prospective clinical trials that reported irAEs, which were clearly identified as “AEs of special interest,” “immune-mediated adverse events,” or “selected treatment-related adverse events of special interest,” but not “treatment-related AEs” or “drug-related AEs.” Additionally, every corresponding author of a potential irAE-related study was e-mailed and asked to provide more information about the irAEs and (2) patients were diagnosed with malignancies that were treated with anti-PD-1/PD-L1 agents. Oncologic therapy prior to anti-PD-1/PD-L1 treatment was acceptable.

The exclusion criteria were: (1) Non-oncologic patients (e.g., hepatitis C virus-related patients) treated with anti-PD-1/PD-L1 agents; (2) Oncologic patients treated with anti-PD-1/PD-L1 agents combined with other treatments simultaneously; (3) Retrospective studies, meeting abstracts, case reports, basic research, reviews, systematic reviews and meta-analysis, letters, editorials, and expert opinions; and (4) Duplicate publications or unpublished studies.

### Data extraction

The titles and abstracts of all studies retrieved were independently reviewed by two authors. Next, the full texts of all potentially eligible studies were assessed. A standardized, pre-piloted form was used to extract relevant information from the included studies.

The primary outcomes for this meta-analysis were incidence and risk ratio (RR) of irAEs and their grade (1–5; recorded according to Version 3 or 4 of the Common Terminology Criteria for Adverse Events of the National Cancer Institute). We considered that Grades ≥3 were evaluated as high grade or severe grade. The secondary outcome was incidence of death due to irAEs. Any discrepancies were solved by discussion. Missing data were requested from the principle investigator by e-mail.

### Quality assessment

Two independent investigators assessed the risk of bias for the included studies according to the Cochrane Handbook for Systematic Reviews of Interventions (Higgins et al., 2011). The following components were assessed: sequence generation, allocation concealment, blinding, completeness of outcome data, incomplete outcome data, and other sources of bias. Disagreements were resolved by discussion among investigators until a consensus was reached.

### Statistical analysis

The incidence and RR of irAEs were estimated for the included studies in this meta-analysis. We pooled the incidence of irAEs in malignancies treated with anti-PD/PD-L1 agents. Heterogeneity between studies was assessed by Q test and I^2^ statistics. If the I^2^ value was less than 50%, the meta-analysis was performed using the fixed-effects model. Otherwise, the random-effects model was adopted. Potential publication bias was examined by funnel plots and Egger's test. Incidence was calculated using R software [R version 3.3.2 (2016-10-31)] with package Meta and Metaprop function. RR was calculated using Review Manager 5.3 (Nordic Cochrane Centre, Copenhagen, Denmark).

## Results

### Literature search

**Figure 1:**
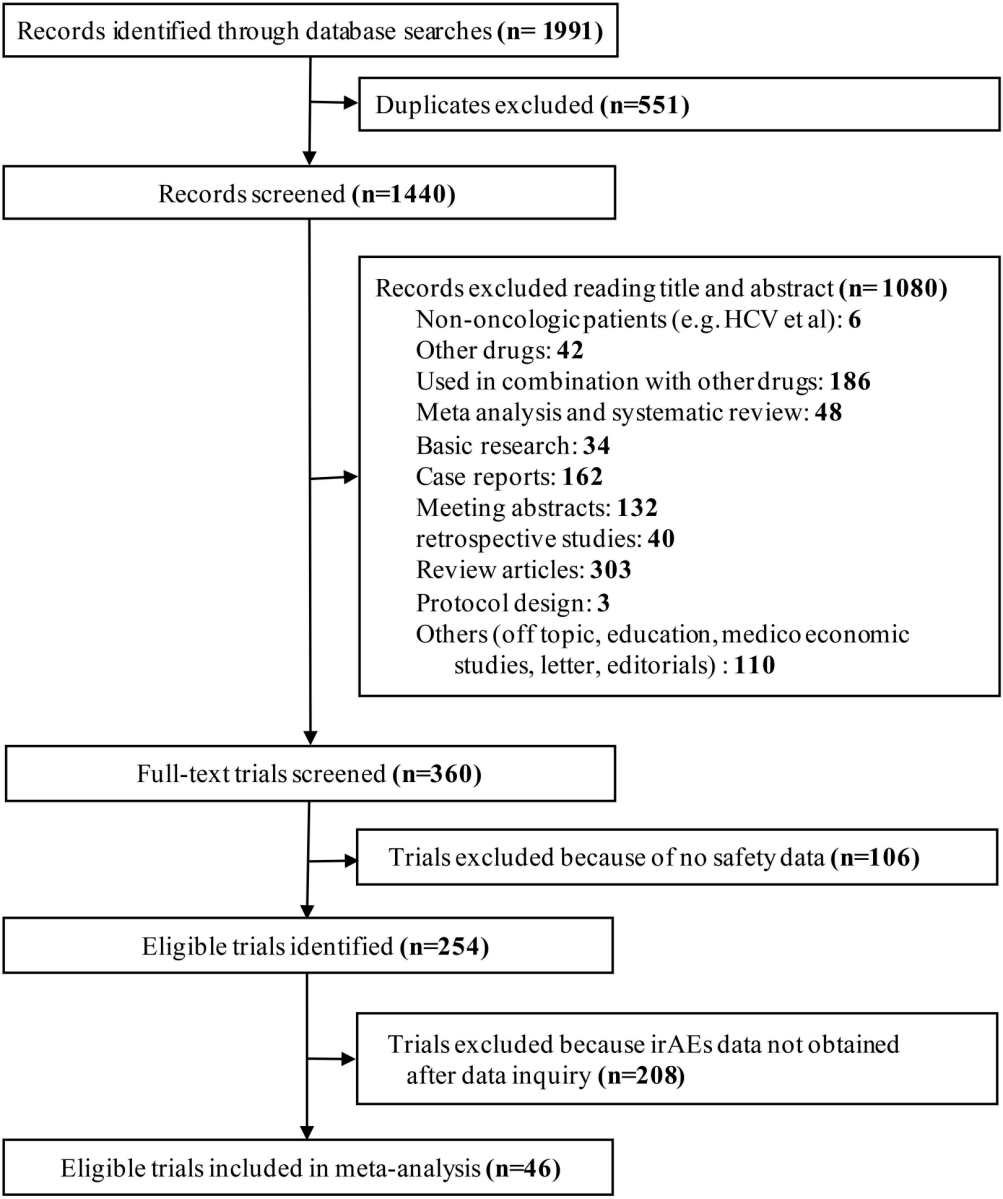
Flow diagram for identification and selection of studies included in the meta-analysis.

Our search strategy identified 1,991 potential articles. Five-hundred and 51 studies were excluded owing to duplicates. The remaining articles were screened for titles and abstracts, and 1080 articles were removed based on our inclusion or exclusion criteria. Furthermore, 314 studies were dropped because they did not contain our data of interest. Finally, 46 studies were included in our meta-analysis. The study selection is shown in Figure 1.

### Study characteristics

The detailed information of the clinical trials is presented in Table 1. Overall, 46 trials comprising 12,808 oncologic patients treated with anti-PD-1/PD-L1 agents were included in this meta-analysis (Table 1) (Brahmer et al., 2010, 2012, 2015; Topalian et al., 2012, 2014; Robert et al., 2014, 2015a,b; Borghaei et al., 2015; Garon et al., 2015; Gettinger et al., 2015, 2016; Hamanishi et al., 2015; Larkin et al., 2015; McDermott et al., 2015, 2016; Motzer et al., 2015; Patnaik et al., 2015; Ribas et al., 2015, 2016; Rizvi et al., 2015; Weber et al., 2015, 2016; Balar et al., 2016; Chatterjee et al., 2016; Choueiri et al., 2016; Fehrenbacher et al., 2016; Ferris et al., 2016; Herbst et al., 2016; Hua et al., 2016; Kaufman et al., 2016; Lesokhin et al., 2016; Massard et al., 2016; Muro et al., 2016; Nanda et al., 2016; Reck et al., 2016; Rosenberg et al., 2016; Seiwert et al., 2016; Shimizu et al., 2016; Younes et al., 2016; Bellmunt et al., 2017; Hui et al., 2017; Morris et al., 2017; Plimack et al., 2017; Sharma et al., 2017). Among these studies, there were 35 phase I/II clinical trials and 11 phase III clinical trials. Nine percent (4/46) of the studies were blind and 13% (6/46) were single-arm. There were 23 randomized trials and 18 multicentric studies. There were 21 studies involving nivolumab treatment (4,856 patients), 17 studies involving pembrolizumab treatment (5,581 patients), 5 trials involving atezolizumab treatment (2,015 patients), one trial involving durvalumab treatment (61 patients), and one trial involving avelumab treatment (88 patients).

**Table 1:** Characteristics of studies included for meta-analysis.

**Study**	**Design**	**Cancer**	**Size**	**Target**	**Drug**	**Dose(mg/kg)**	**CTC for AE version^c^**
Brahmer et al., 2010	Non-Randomized, open label, phase I	Melanoma, NSCLC^b^, RCC^b^, CRC^b^, and prostate cancer	39	PD-1	Nivolumab (MDX-1106)^a^	0.3; 1; 3; 10	n/a
Topalian et al., 2012	Non-Randomized, open label, phase I	Melanoma, NSCLC, RCC, CRC, and prostate cancer	296	PD-1	Nivolumab (MDX-1106)	0.1; 0.3; 1; 3; 10	3
Topalian et al., 2014	Non-Randomized, open label, phase I	Melanoma	107	PD-1	Nivolumab	1; 3; 10	3
Brahmer et al., 2015	Randomized, open label, phase III	NSCLC	272	PD-1	Nivolumab	3	4
Gettinger et al., 2015	Non-Randomized, open label, phase I	NSCLC	129	PD-1	Nivolumab	1; 3; 10	3
Hamanishi et al., 2015	Open label, single center, phase II	Ovarian, Peritoneal	20	PD-1	Nivolumab	1; 3	4
Larkin et al., 2015	Randomized, double-blind, phase III	Melanoma	945	PD-1	Nivolumab	3	4
McDermott et al., 2015	Non-Randomized, open label, phase I	RCC	34	PD-1	Nivolumab	1; 10	3
Motzer et al., 2015	Randomized, double-blind, phase II	RCC	168	PD-1	Nivolumab	0.3; 2; 10	4
Rizvi et al., 2015	Open label, single-arm, phase II	NSCLC	117	PD-1	Nivolumab	3	4
Robert et al., 2015a	Randomized, double-blind, phase III	Melanoma	418	PD-1	Nivolumab	3	4
Weber et al., 2015	Randomized, open label, phase III	Melanoma	631	PD-1	Nivolumab	3	4
Choueiri et al., 2016	Randomized, open label, phase Ib	RCC	91	PD-1	Nivolumab	0.3; 2; 10	4
Gettinger et al., 2016	Randomized, multi-arm, phase I	NSCLC	52	PD-1	Nivolumab	3	4
Lesokhin et al., 2016	Randomized, open label, phase I	Hematologic Malignancy	81	PD-1	Nivolumab	1; 3	4
Younes et al., 2016	Open label, single-arm, phase II	Hodgkin lymphoma	80	PD-1	Nivolumab	3	4
Ferris et al., 2016	Randomized, open label, phase III	NSCLC	361	PD-1	Nivolumab	3	4
Weber et al., 2016	Non-Randomized, phase I/ II	Melanoma	126	PD-1	Nivolumab	3	4
Borghaei et al., 2015	Randomized, open label, phase III	NSCLC	582	PD-1	Nivolumab	3	4
Sharma et al., 2017	Single-arm, phase II	Urothelial	270	PD-1	Nivolumab	3	4
Morris et al., 2017	Single-arm, phase II	SCCA^b^	37	PD-1	Nivolumab	3	4
Robert et al., 2014	Randomized, phase I	Melanoma	173	PD-1	Pembrolizumab	2; 10	4
Patnaik et al., 2015	Randomized, open label, phase I	Multiple solid tumors^d^	32	PD-1	Pembrolizumab	1; 2; 3; 10	4
Chatterjee et al., 2016	Randomized, open label, phase I	NSCLC	550	PD-1	Pembrolizumab	2; 10	4
Herbst et al., 2016	Randomized, open label, phase II/III	NSCLC	1034	PD-1	Pembrolizumab	2; 10	4
Reck et al., 2016	Randomized, open label, phase III	NSCLC	305	PD-1	Pembrolizumab	n/a	4
Ribas et al., 2016	Randomized, open label, phase I	Melanoma	655	PD-1	Pembrolizumab	2; 10	4
Seiwert et al., 2016	Non-Randomized, open label, phase I	HNSCC^b^	60	PD-1	Pembrolizumab	10	4
Shimizu et al., 2016	Non-Randomized, open label, phase I	Melanoma, NSCLC and breast cancer	10	PD-1	Pembrolizumab	2; 10	4
Hua et al., 2016	Prospective observational study, phase I	Melanoma	67	PD-1	Pembrolizumab	n/a	4
Muro et al., 2016	Non-Randomized, open label, phase Ib	Gastric cancer	39	PD-1	Pembrolizumab	10	4
Garon et al., 2015	Randomized, phase I	NSCLC	495	PD-1	Pembrolizumab	2; 10	4
Ribas et al., 2015	Randomized, controlled, phase II	Melanoma	540	PD-1	Pembrolizumab	2	4
Nanda et al., 2016	Non-Randomized, phase Ib	Breast	111	PD-1	Pembrolizumab	10	4
Robert et al., 2015b	Randomized, controlled, double-blind, three-arm phase III	Melanoma	834	PD-1	Pembrolizumab	10	4
Plimack et al., 2017	Non-Randomized, phase Ib	Urothelial	33	PD-1	Pembrolizumab	10	4
Hui et al., 2017	Randomized, open label, phase I	NSCLC	101	PD-1	Pembrolizumab	2; 10	4
Bellmunt et al., 2017	Randomized, open label, phase III	Urothelial	542	PD-1	Pembrolizumab	200^f^	4
Brahmer et al., 2012	Non-Randomized, open label, phase I	Multiple solid tumors^e^	207	PD-L1	BMS-936559	0.3; 1; 3; 10	3
McDermott et al., 2016	Open label, phase I	RCC	70	PD-L1	Atezolizumab	3; 10; 15; 20; 1200^f^	4
Rosenberg et al., 2016	Open label, single-arm, phase II	Urothelial	310	PD-L1	Atezolizumab	1200 ^f^	4
Balar et al., 2016	Open label, single-arm, phase II	Urothelial	123	PD-L1	Atezolizumab	1200^f^	4
Rittmeyer et al., 2017	Randomized, open label, phase III	NSCLC	1225	PD-L1	Atezolizumab	1200^f^	4
Fehrenbacher et al., 2016	Randomized, open label, phase II	NSCLC	287	PD-L1	Atezolizumab	1200^f^	4
Massard et al., 2016	Open label, phase I/II	Urothelial Bladder carcinoma	61	PD-L1	Durvalumab	10	4
Kaufman et al., 2016	Open label, phase II	Merkel cell carcinoma	88	PD-L1	Avelumab	10	4

a *Nivolumab, MDX-1106 = BMS-936558; MDX-1105 = BMS-936559; Pembrolizumab, MK-3475 = lambrolizumab; Atezolizumab = PCD4989g = NCT01375842; Durvalumab = MEDI473*.

b *RCC, renal cell carcinoma; NSCLC, non-small-cell lung cancer; HNSCC, head and neck squamous cell carcinoma; SCCA, squamous cell carcinoma of the anal canal; CRC, colorectal cancer*.

c *CTC for AE version, Common Terminology Criteria for Adverse Events version; n/a, non-available*.

d *Include patients with melanoma, carcinoid, colorectal, prostate, Merkel cell, NSCLC, adenocarcinoma, kaposi sarcoma, pancreatic, squamous cell lung cancer, soft tissue sarcoma, peripheral nerve sheath tumor*.

e *Include patients with melanoma, NSCLC, RCC, ovary, CRC, pancreatic, gastric and breast*.

f *Exposure at a fixed dose every experimental cycle*.

### Global incidence of irAEs

**Figure 2:**
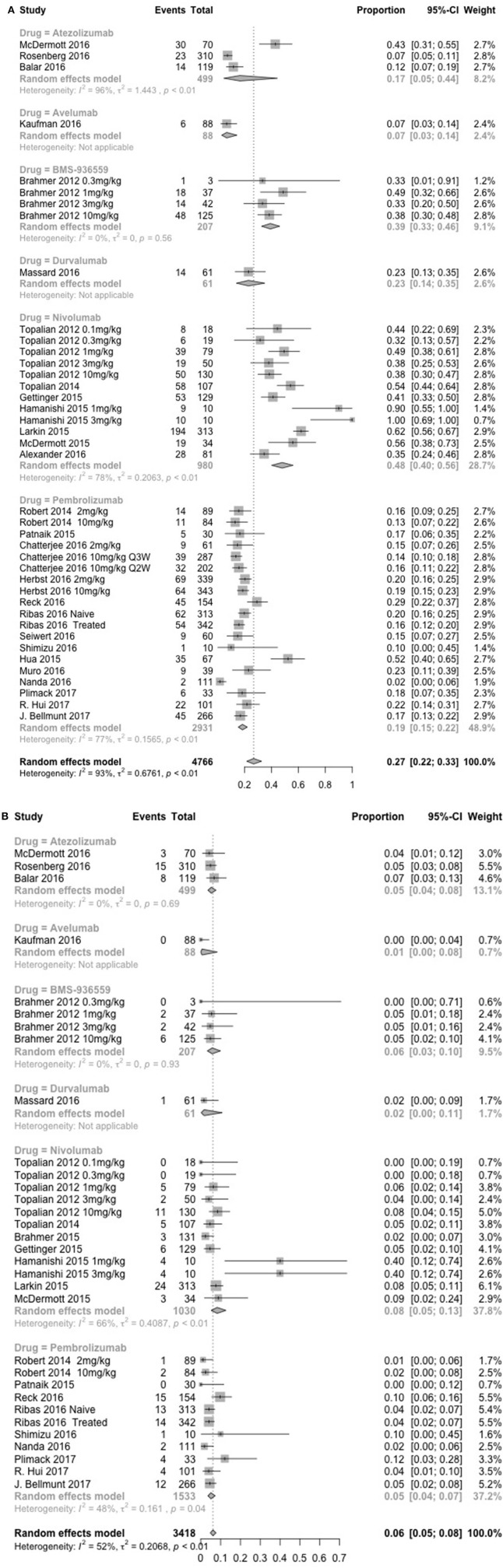
Incidence of global immune-related adverse events (irAEs) with nivolumab (1 and 3 mg/kg), pembrolizumab (2 mg/kg, 10 mg/kg, 200 mg), atezolizumab (1,200 mg) at all dosage, any grade **(A)**, and severe grade **(B)**.

The global incidence of irAEs of any grade and severe grade in patients treated with anti-PD-1/PD-L1 agents was 26.82% (95% CI, 21.73–32.61; I^2^, 92.80) and 6.10% (95% CI, 4.85–7.64; I^2^, 52.00), respectively (Figure S1). The incidence of any grade of irAEs was 18.50% (95% CI, 15.41–22.06; I^2^, 77.00) with pembrolizumab and 16.67% (95% CI, 4.75–44.50; I^2^, 96.00) with atezolizumab, which was lower than the incidence of irAEs with nivolumab (48.00%, 95% CI, 40.13–55.98; I^2^, 78.50). However, there were only slight differences in the incidence of severe grade irAEs, which was 8.25% (95% CI, 5.27–12.69; I^2^, 66.40) with nivolumab, 5.10% (95% CI, 3.58–7.22; I^2^, 48.40) with pembrolizumab, and 5.28% (95% CI, 3.62–7.64; I^2^, 0.00) with atezolizumab (Figure 2).

### Organ-specific irAEs

We analyzed the incidence of irAEs associated with PD-1 blockade involving the skin, endocrine system, gastrointestinal tract, liver, kidney, and lung. The most common irAEs of all grades caused by nivolumab involved the skin, followed by the gastrointestinal tract, endocrine system, liver, lung, and kidney. The severe grade irAEs caused by nivolumab mostly occurred in the gastrointestinal tract and liver. However, we found it difficult to draw robust conclusions owing to limited data on pembrolizumab. The detailed information of organ-specific irAEs is presented in Tables S3–S8.

#### Skin

The most frequent irAEs during treatment with nivolumab were pruritus and rash, with an incidence of approximately 13%. However, pembrolizumab tended to have a lower incidence, less than 5% of any cutaneous irAEs of all grade. Notably, severe grade incidence of any irAEs was ~2% with nivolumab, pembrolizumab, and BMS-936559 (Table S9).

#### Endocrine system

The most common endocrine disorder associated with PD-1 blockade with nivolumab and pembrolizumab was hypothyroidism. PD-L1 blockade was associated with nearly the same incidence of irAEs for every endocrine disease. For all the drugs, the incidence of severe grade irAEs occurring in the endocrine system was ≤2% (Table S10).

#### Gastrointestinal tract

Diarrhea was the most frequent irAEs in patients treated with nivolumab or BMS-936559, and its incidence was approximately 10–13%. Pembrolizumab was likely to have a relatively low risk of diarrhea of any grade, and its incidence was less than 4%. In addition, the incidence of high-grade colitis was ~1% regardless of the treatment type. Pancreatitis was rare, with an incidence of less than 1% for either all grade or severe grade across all drug types (Table S11).

#### Liver

The incidence of increased aspartate aminotransferase (AST) or alanine aminotransferase (ALT) levels ranged from 4 to 5% with nivolumab treatment. This incidence of increased AST or ALT levels was around 2% in patients treated with pembrolizumab or atezolizumab. However, the incidence of elevated AST/ALT/blood bilirubin and hepatitis was ≤2% regardless of treatment type (Table S12).

#### Pulmonary

Pneumonitis was the most common irAE related to the lung. The incidence of pneumonitis of any grade was approximately 3% and that of severe grade was approximately 1% during treatment with nivolumab or pembrolizumab. Treatment with atezolizumab was less likely to be associated with pneumonitis, with an incidence of ~1% for any grade and <1% for severe grade (Table S13).

#### Kidney

The incidence of any grade of nephritis was very low (less than 1%) regardless of the treatment type. There were reports of acute renal failure and renal failure only with nivolumab treatment, although the incidence was only ~2% for all grade and ~1% for severe grade (Table S14).

### Tumor-specific irAEs

We analyzed the incidence of irAEs with regard to the tumor being treated, including melanomas, NSCLCs, RCCs, and urothelial tumors. The highest incidence of any irAE associated with nivolumab and pembrolizumab occurred during the treatment of melanomas and NSCLCs, respectively. In contrast, the most frequent severe grade irAEs occurred with nivolumab treatment of melanomas.

#### Melanoma

The global incidence of all grade irAEs was 59.06% (95% CI, 51.53–66.18; I^2^, 50.00) with nivolumab and 21.33% (95% CI, 12.94–33.10; I^2^, 90.80) with pembrolizumab. The incidence of severe grade irAEs was low, ranging from 6.94% (95% CI, 4.69–10.16; I^2^, 8.3) with nivolumab to 3.80% (95% CI, 2.67–5.39; I^2^, 0.0%) with pembrolizumab (Figure S2).

The most frequent irAEs in patients treated with nivolumab were pruritus, followed by diarrhea and rash. Pembrolizumab treatment was mostly associated with hypothyroidism. In patients treated with nivolumab, the most frequent severe irAE was AST/ALT increase, with an incidence of ~3%, while the other irAEs were rare (<1%). The severe irAEs associated with pembrolizumab administration were maculopapular rash, colitis, increased AST, and hepatitis, occurring at 1–2% (Tables S15–S20).

#### NSCLC

The most common irAEs associated with nivolumab were rash and diarrhea, and those associated with pembrolizumab were hypothyroidism and hyperthyroidism. The incidence of any grade of irAEs with pembrolizumab was higher than that with nivolumab. Severe grade irAEs were rare (Tables S21–S26).

#### RCC

Diarrhea and colitis were the most frequent any grade irAEs and severe grade irAEs, respectively, associated with nivolumab treatment (Tables S27–S32).

#### Urothelial carcinomas

The global incidence of any grade irAEs in urothelial cancers treated with pembrolizumab and atezolizumab was 17.06% (95% CI, 13.21; 21.76; I^2^, 0.00) and 9.09% (95% CI, 5, 75; 14.09; I^2^, 50.60), respectively. The number of studies providing statistics on organ-specific irAEs in patients with urothelial cancer was relatively small. From the limited cases, we found that the incidence of all grade irAEs and severe grade irAEs in specific organs were very rare, with incidence of ≤ 1–3% (Table S33).

### Dose-dependent analysis

The incidence of all grade irAEs in patients treated with 3 mg/kg nivolumab was 58.08% (95% CI, 34.05–78.81; I^2^, 84.80), while that in patients treated with 1 mg/kg nivolumab was 70.00% (95% CI, 21.76–95.14; I^2^, 76.50). This dose-dependent effect was also observed in severe grade irAEs, with incidence of 7.84% (95% CI, 2.62–21.22; I^2^, 81.30) at 3 mg/kg and 16.88% (95% CI, 2.11–65.64; I^2^, 88.00) at 1 mg/kg (Table S1). Owing to limited data, we could not calculate the RR for nivolumab. However, we hypothesized that dose would not affect the development of irAEs as the 95% CI overlapped greatly.

In the treatment with pembrolizumab, the incidence of all grade irAEs was 18.93% (95% CI, 15.69–22.67; I^2^, 0.00) at 2 mg/kg, while that at 10 mg/kg was 15.16% (95% CI, 11.85–19.20; I^2^, 56.00). It was also observed that the incidence of severe grade irAEs was 1.12% (95% CI, 0.16–7.54) with 2 mg/kg pembrolizumab and 4.07% (95% CI, 1.10–13.91; I^2^, 69.70) with 10 mg/kg pembrolizumab (Table S2). The RR of developing any grade of global irAEs with pembrolizumab at 10 mg/kg vs. that at 2 mg/kg was not statistically different [RR, 0.91 (0.70, 1.17); *P* = 0.46; Figure S3].

### Death related to irAEs

Deaths due to irAEs were reported in 33 studies comprising 5,090 patients. Death occurred in nine of 5,090 patients, with an incidence of 0.74 (95% CI, 00.44; 1.25; I^2^, 38.20). Four patients died owing to pembrolizumab treatment, with an incidence of 0.89 (95 % CI, 00.49; 1.62; I^2^, 0.00). Five patients died following nivolumab treatment, and four of the deaths were due to pneumonitis. The incidence of deaths following nivolumab treatment was 0.77 (95% CI, 00.32; 1.87; I^2^, 56.20). Drug/treatment-related deaths and studies not reporting deaths were not included in this meta-analysis.

### Quality assessment and publication bias

Risk of bias graphs and risk of bias summaries from Review Manager 5.3 were used to evaluate the methodological qualities of the studies. Blinding of participants and personnel was evaluated as a low risk item because many studies were dose-escalation and single-arm trials. The overall risk of bias was evaluated as low risk. Therefore, the quality of the studies was satisfactory (Figure S4).

The funnel plot and Egger's test for publication bias showed a symmetric distribution of trials on either side of the funnel. The Begg's test and Egger's test (*P* = 0.7355) also indicated that no significant publication bias existed in this meta-analysis (Figure S5).

## Discussion

In the present study, we systematically characterized the occurrence of irAEs in oncologic patients treated with anti-PD-1/PD-L1 agents according to different targets, drug types, drug dose, organ-specificity, and the tumor type treated. Although immune-check point inhibitors improved clinical outcomes in oncology, their irAEs should not be neglected (Bertrand et al., 2015; Michot et al., 2016; Costa et al., 2017; Tie et al., 2017). A previous meta-analysis indicated a high risk of irAEs with anti-CTLA-4 treatment, with incidence of 72% (95% CI, 65–79%) for all grade irAEs and 24% (95% CI, 18–30%) for high grade irAEs, and the occurrence of irAEs following anti-CTLA-4 treatment was dose-dependent (Bertrand et al., 2015). However, we found a much lower incidence of irAEs in patients treated with any of the anti-PD-1/PD-L1 agents, regardless of the drug type or dose. This indicates better tolerance of PD-1/PD-L1 inhibitors in cancer patients. Moreover, severe grade irAEs caused by any anti-PD-1/PD-L1 agent had a very low incidence, ranging from 5 to 8%. This helped in maximizing patient safety and treatment duration, because serious irAEs may result in the discontinuation of anti-PD-1/PD-L1 treatment in patients (Kumar et al., 2017).

A wide spectrum of irAEs was induced by all anti-PD-1/PD-L1 agents, including those involving the skin, gastrointestinal tract, lung, kidney, and liver. Michot et al. suggested that irAEs of grade I-II mainly affected the skin and gastrointestinal tract, and severe grade irAEs mostly occurred within the digestive system (Michot et al., 2016). We also found organ-specific irAEs associated with PD-1/PD-L1 blockade; however, our results indicated that these organ-specific characteristics were related to drug types. During nivolumab treatment, the most common irAEs of all grades were skin- (e.g., pruritus, rash) and gastrointestinal tract-related (e.g., diarrhea), with an incidence of approximately 13%. In contrast, during pembrolizumab treatment, the irAE with the highest incidence (~8%) was hypothyroidism (Tables S3–14). Additionally, the incidence of irAEs caused by PD-1 blockade was tumor-specific. Pneumonitis of all grades more frequently occurred during NSCLC and RCC treatment than during melanoma treatment. We also found a higher incidence of pneumonitis during NSCLC (4.15%, 95% CI, 3.36–5.12%) and RCC (7.19%, 95% CI, 4.54–11.21%) treatments than during melanoma treatment (2.18%, 95% CI, 1.67–2.84%; Tables S19, S25, and S31). Moreover, the incidence of all grade pruritus and diarrhea was lower in NSCLC than in melanoma and RCC during nivolumab treatment (Tables S15, S17, S21, S23, S27, and S29), confirming that the irAEs were tumor-specific. Our results correspond well with those published previously (Nishino et al., 2016) and are arguably more convincing owing to the larger number of studies involved.

In addition to the irAEs caused by anti-PD-1/PD-L1 agents, the response rate of this immunotherapy was another concern (Gibney et al., 2016; Topalian et al., 2016). Fortunately, genomic sequencing, RNA sequencing, and whole exome sequencing were of great importance in identifying predictive biomarkers for anti-PD-1/PD-L1 agent responses Gibney et al., 2016; Topalian et al., 2016; Hugo et al., 2017. Similarly, we could utilize the methods above to identify the genes associated with a higher risk of irAEs, especially with severe grade irAEs, which can sometimes be fatal. We conclude that the pattern of irAE occurrence was dependent on drug type, tumor type, irAE grades, target organ, and the particular irAE. However, the dataset in our analysis could ideally have been larger. As there are many ongoing studies on anti-PD-1/PD-L1 agents, we recommend that the factors related to irAEs be recorded in these clinical trials.

## Limitations

There were some limitations in our study. First, the variability of clinical settings was high. Adopting the random-effects model was an important step to control variability. In addition, the main source of variability was significantly reduced when subgroup analysis was performed based on irAE grades, organs, drug types, and tumor types. These results provide clinical value for physicians to be wary about the occurrence of irAEs when treating specific tumors with different drugs. Second, the number of studies and patients were limited in the subgroup analysis performed based on drug type, organ-specific irAEs, particular irAEs, and tumor type. Third, some of the included studies were not random clinical trials, introducing some bias in our analysis. Last, the incidence of irAEs was not clear in patients with preexisting autoimmune diseases according to our analysis. Because all the included studies excluded patients with active autoimmune disease or a documented history of autoimmune disease or syndrome that requires systemic steroids or immunosuppressive agents, except vitiligo or resolved childhood asthma/atopy. Therefore, the safety of PD-1 inhibitors in patients with pre-existing autoimmune diseases needed to be assessed further in experimental or clinical studies.

## Conclusion

In conclusion, our meta-analysis provided a statistical overview of irAEs in oncologic patients treated with anti-PD-1/PD-L1 agents. The pattern of irAE occurrence was organ-specific and related to the drug and tumor types.

## Author contributions

Conception and design: CY and NL; Collection and assembly of data: PW and YC; Data analysis and interpretation: All authors; Manuscript writing: All authors; Final approval of manuscript: All authors; Accountable for all aspects of the work: All authors.

### Conflict of interest statement

The authors declare that the research was conducted in the absence of any commercial or financial relationships that could be construed as a potential conflict of interest.
