# Supplementary material for: Immune-Related Adverse Events Associated with Anti-PD-1/PD-L1 Treatment for Malignancies: A Meta-Analysis
Source: Front Pharmacol. 2017 Oct 18;8:730. doi: 10.3389/fphar.2017.00730 (PMC5651530; doi:10.3389/fphar.2017.00730)
Supplement: Supplementary file 1 [file Table2.docx]

**Supplementary Appendix: Immune related adverse events associated with anti-PD1/PD-L1 treatment for malignancies: a meta-analysis. Wang et al.**

**Contents**

**Supplementary Tables**...............................................................................................................................................................................................................1

1. **Incidence of irAEs in all cancer patients**..................................................................................................................................................................2

Table S1 Global incidence of irAEs with nivolumab 1mg/kg, 3mg/kg, any grade and severe grade in all cancer patients...................................................2

Table S2 Global incidence of irAEs with pembrolizumab 2mg/kg, 10mg/kg (Q2W, Q3W), 200mg, any grade and severe grade in all cancer patients….2

Table S3 Incidence of cutaneous irAEs with nivolumab 0.3mg/kg, 2mg/kg and 3mg/kg any grade and severe grade in all cancer patients........................2

Table S4 Incidence of endocrinologic irAEs with nivolumab 0.3mg/kg, 2mg/kg and 3mg/kg any grade and severe grade in all cancer patients................2

Table S5 Incidence of gastrointestinal irAEs with nivolumab 0.3mg/kg, 2mg/kg and 3mg/kg any grade and severe grade in all cancer patients................2

Table S6 Incidence of hepatic irAEs with nivolumab 0.3mg/kg, 2mg/kg and 3mg/kg any grade and severe grade in all cancer patients.............................3

Table S7 Incidence of pulmonary irAEs with nivolumab 0.3mg/kg, 2mg/kg and 3mg/kg any grade and severe grade in all cancer patients.......................3

Table S8 Incidence of renal irAEs with nivolumab 0.3mg/kg, 2mg/kg and 3mg/kg any grade and severe grade in all cancer patients.................................3

Table S9 Particular incidence of cutaneous irAEs in all cancer patients..................................................................................................................................3

Table S10 Particular incidence of endocrinologic irAEs in all cancer patients........................................................................................................................5

Table S11 Particular incidence of gastrointestinal irAEs in all cancer patients................................................................................................................ .......6

Table S12 Particular incidence of hepatic irAEs in all cancer patients...................................................................................................................... ...... .......7

Table S13 Particular incidence of pulmonary irAEs in all cancer patients........................................................................................................................ ......8

Table S14 Particular incidence of renal irAEs in all cancer patients.................................................................................................................................. ......9

1. **Incidence of irAEs in melanomas patients**............................................................................................................................................................ ...... .......9

Table S15 Particular incidence of cutaneous irAEs in melanomas patients.............................................................................................................................10

Table S16 Particular incidence of endocrinologic irAEs in melanomas patients.....................................................................................................................10

Table S17 Particular incidence of gastrointestinal irAEs in melanomas patients.................................................................................................................... 10

Table S18 Particular incidence of hepatic irAEs in melanomas patients..................................................................................................................................11

Table S19 Particular incidence of pulmonary irAEs in melanomas patients............................................................................................................................12

Table S20 Particular incidence of renal irAEs in melanomas patients......................................................................................................................................12

1. **Incidence of irAEs in NSCLC patients**................................................................................................................................................................................12

Table S21 Particular incidence of cutaneous irAEs with nivolumab (3 mg/kg), any grade and severe grade in NSCLC patients...........................................12

Table S22 Particular incidence of endocrinologic irAEs any grade and severe grade in NSCLC patients...............................................................................13

Table S23 Particular incidence of gastrointestinal irAEs any grade and severe grade in NSCLC patients...............................................................................14

Table S24 Particular incidence of hepatic irAEs any grade and severe grade in NSCLC patients............................................................................................14

Table S25 Particular incidence of pulmonary irAEs any grade and severe grade in NSCLC patients......................................................................................15

Table S26 Particular incidence of renal irAEs with nivolumab (3 mg/kg), any grade and severe grade in NSCLC patients.................................................. 15

1. **Incidence of irAEs in RCC patients**......................................................................................................................................................................................15

Table S27 Particular incidence of cutaneous irAEs with nivolumab, any grade and severe grade in RCC patients.................................................................15

Table S28 Particular incidence of endocrinologic irAEs with nivolumab, any grade and severe grade in RCC patients.........................................................15

Table S29 Particular incidence of gastrointestinal irAEs with nivolumab, any grade and severe grade in RCC patients.........................................................16

Table S30 Particular incidence of hepatic irAEs with nivolumab, any grade and severe grade in RCC patients......................................................................16

Table S31 Particular incidence of pulmonary irAEs with nivolumab, any grade and severe grade in RCC patients................................................................16

Table S32 Particular incidence of renal irAEs with nivolumab, any grade and severe grade in RCC patients.........................................................................17

1. **Incidence of irAEs in urothelial carcinomas patients**.................................................................................................................................. ...... ...... .........17

Table S33 Incidence of irAEs with pembrolizumab and atezolizumab, any grade and severe grade in urothelial carcinomas patients....................................17

**Supplementary Tables**

1. **Incidence of irAEs in all cancer patients**

**Table S1** Incidence of global irAEs with nivolumab 1mg/kg, 3mg/kg, any grade and severe grade in all cancer patients

| Nivolumab | Any Grade irAEs | | | Severe Grade irAEs | | |
| --- | --- | --- | --- | --- | --- | --- |
|  | Studies/Patients | Incidence | I^2^ | Studies/Patients | Incidence | I^2^ |
| All dosage | 12/980 | 0.4800 [0.4013; 0.5598] | 78.5% | 12/1030 | 0.0825 [0.0527; 0.1269] | 66.4% |
| 1 mg/kg | 2/89 | 0.7000 [0.2176; 0.9514] | 76.50% | 2/89 | 0.1688 [0.0211; 0.6564] | 88.00% |
| 3 mg/kg | 3/373 | 0.5808 [0.3405; 0.7881] | 84.80% | 4/504 | 0.0784 [0.0262; 0.2122] | 81.30% |

**Table S2** Incidence of global irAEs with pembrolizumab 2mg/kg, 10mg/kg (Q2W, Q3W), 200mg, any grade and severe grade in all cancer patients

| Pembrolizumab | Any Grade irAEs | | | Severe Grade irAEs | | |
| --- | --- | --- | --- | --- | --- | --- |
|  | Studies/Patients | Incidence | I^2^ | Studies/Patients | Incidence | I^2^ |
| All dosage | 19/2931 | 0.1850 [0.1541; 0.2206] | 77.00% | 11/1533 | 0.0510 [0.0358; 0.0722] | 48.40% |
| 2 mg/kg | 3/489 | 0.1893 [0.1569; 0.2267] | 0.00% | 1/89 |  |  |
| 10 mg/kg | 8/1159 | 0.1516 [0.1185; 0.1920] | 56.00% | 3/228 | 0.0407 [0.0110; 0.1391] | 69.70% |
| Q2W | 5/445 | 0.1387 [0.0861; 0.2158] | 60.10% | 2/144 | 0.0505 [0.0073; 0.2769] | 80.50% |
| Q3W | 3/714 | 0.1566 [0.1219; 0.1989] | 43.70% | 1/84 |  |  |
| 200mg | 2/420 | 0.2242 [0.1263; 0.3662] | 88.40% | 2/420 | 0.0672 [0.0310; 0.1392] | 76.40% |

**Table S3** Incidence of cutaneous irAEs with nivolumab 0.3mg/kg, 2mg/kg and 3mg/kg any grade and severe grade in all cancer patients

| Nivolumab | Any Grade irAEs | | Severe Grade irAEs | | | | |
| --- | --- | --- | --- | --- | --- | --- | --- |
|  | Studies/Patients | Incidence | I^2^ | Studies/Patients | Incidence | I^2^ |  |
| All dosage | 19/2249 | 0.2547 [0.2050; 0.3116] | 86.40% | 18/2169 | 0.0184 [0.0128; 0.0261] | 0.00% |  |
| 0.3 mg/kg | 2/82 | 0.2941 [0.1456; 0.5046] | 66.00% | 2/82 | 0.0133 [0.0019; 0.0887] | 0.00% |  |
| 2 mg/kg | 2/76 | 0.2237 [0.1438; 0.3308] | 0.00% | 2/76 | 0.0401 [0.0130; 0.1173] | 0.00% |  |
| 3 mg/kg | 9/1673 | 0.2393 [0.1694; 0.3266] | 92.60% | 8/1593 | 0.0164 [0.0103; 0.0261] | 12.50% |  |

**Table S4** Incidence of endocrinologic irAEs with nivolumab 0.3mg/kg, 2mg/kg and 3mg/kg any grade and severe grade in all cancer patients

| Nivolumab | Any Grade irAEs | | | Severe Grade irAEs | | |
| --- | --- | --- | --- | --- | --- | --- |
|  | Studies/Patients | Incidence | I^2^ | Studies/Patients | Incidence | I^2^ |
| All dosage | 18/2168 | 0.1024 [0.0832; 0.1253] | 53.10% | 17/2088 | 0.0136 [0.0088; 0.0209] | 0.00% |
| 0.3 mg/kg | 2/82 | 0.0617 [0.0259; 0.1399] | 0.00% | 2/82 | 0.0133 [0.0019; 0.0887] | 0.00% |
| 2 mg/kg | 2/76 | 0.1189 [0.0630; 0.2130] | 0.00% | 2/76 | 0.0401 [0.0130; 0.1173] | 0.00% |
| 3 mg/kg | 9/1673 | 0.0975 [0.0723; 0.1303] | 72.30% | 8/1593 | 0.0078 [0.0043; 0.0141] | 0.00% |

**Table S5** Incidence of gastrointestinal irAEs with nivolumab 0.3mg/kg, 2mg/kg and 3mg/kg any grade and severe grade in all cancer patients

| Nivolumab | Any Grade irAEs | | | Severe Grade irAEs | | |
| --- | --- | --- | --- | --- | --- | --- |
|  | Studies/Patients | Incidence | I^2^ | Studies/Patients | Incidence | I^2^ |
| All dosage | 19/2249 | 0.1360 [0.1091; 0.1684] | 71.20% | 18/2169 | 0.0241 [0.0162; 0.0357] | 31.60% |
| 0.3 mg/kg | 2/82 | 0.0990 [0.0261; 0.3106] | 68.40% | 2/82 | 0.0133 [0.0019; 0.0887] | 0.00% |
| 2 mg/kg | 2/76 | 0.1346 [0.0739; 0.2328] | 0.00% | 2/76 | 0.0258 [0.0065; 0.0972] | 0.00% |
| 3 mg/kg | 9/1673 | 0.1249 [0.0917; 0.1678] | 80.50% | 8/1593 | 0.0202 [0.0140; 0.0291] | 0.00% |

**Table S6** Incidence of hepatic irAEs with nivolumab 0.3mg/kg, 2mg/kg and 3mg/kg any grade and severe grade in all cancer patients

| Nivolumab | Any Grade irAEs | | | Severe Grade irAEs | | |
| --- | --- | --- | --- | --- | --- | --- |
|  | Studies/Patients | Incidence | I^2^ | Studies/Patients | Incidence | I^2^ |
| All dosage | 19/2249 | 0.053 [0.0392; 0.0713] | 52.80% | 18/2169 | 0.0227 [0.0146; 0.0353] | 40.90% |
| 0.3 mg/kg | 2/82 | 0.0715 [0.0171; 0.2545] | 61.00% | 2/82 | 0.0449 [0.0083; 0.2078] | 50.30% |
| 2 mg/kg | 2/76 | 0.0957 [0.0462; 0.1878] | 0.00% | 2/76 | 0.0401 [0.0130; 0.1173] | 0.00% |
| 3 mg/kg | 9/1673 | 0.0394 [0.0264; 0.0583] | 55.20% | 8/1593 | 0.0180 [0.0122; 0.0266] | 0.00% |

**Table S7** Incidence of pulmonary irAEs with nivolumab 0.3mg/kg, 2mg/kg and 3mg/kg any grade and severe grade in all cancer patients

| Nivolumab | Any Grade irAEs | | | Severe Grade irAEs | | |
| --- | --- | --- | --- | --- | --- | --- |
|  | Studies/Patients | Incidence | I^2^ | Studies/Patients | Incidence | I^2^ |
| All dosage | 19/2249 | 0.0475 [0.0352; 0.0638] | 49.70% | 18/2169 | 0.0184 [0.0122; 0.0277] | 13.80% |
| 0.3 mg/kg | 2/82 | 0.0488 [0.0184; 0.1230] | 0.00% | 2/82 | 0.0316 [0.0044; 0.1945] | 40.00% |
| 2 mg/kg | 2/76 | 0.0401 [0.0130; 0.1173] | 0.00% | 2/76 | 0.0140 [0.0020; 0.0931] | 0.00% |
| 3 mg/kg | 9/1673 | 0.0294 [0.0210; 0.0412] | 23.20% | 8/1852 | 0.0124 [0.0070; 0.0218] | 15.90% |

**Table S8** Incidence of renal irAEs with nivolumab 0.3mg/kg, 2mg/kg and 3mg/kg any grade and severe grade in all cancer patients

| Nivolumab | Any Grade irAEs | | | Severe Grade irAEs | | |
| --- | --- | --- | --- | --- | --- | --- |
|  | Studies/Patients | Incidence | I^2^ | Studies/Patients | Incidence | I^2^ |
| All dosage | 18/1936 | 0.0366 [0.0233; 0.0573] | 62.70% | 17/1856 | 0.0126 [0.0071; 0.0224] | 24.80% |
| 0.3 mg/kg | 2/82 | 0.0562 [0.0068; 0.3428] | 71.80% | 2/82 | 0.0407 [0.0032; 0.3581] | 66.00% |
| 2 mg/kg | 2/76 | 0.0580 [0.0026; 0.5945] | 78.60% | 2/76 | 0.0140 [0.0020; 0.0931] | 0.00% |
| 3 mg/kg | 8/1360 | 0.0228 [0.0147; 0.0352] | 23.30% | 7/1280 | 0.0064 [0.0032; 0.0131] | 0.00% |

**Table S9** Particular incidence of cutaneous irAEs in all cancer patients

| Target and Drug | Pruritus | | | | | | | Rash | | | | | | |
| --- | --- | --- | --- | --- | --- | --- | --- | --- | --- | --- | --- | --- | --- | --- |
|  | | Studies/Patients | Any Grade | I^2^ | Studies/Patients | Severe Grade | I^2^ | | Studies/Patients | Any Grade | I^2^ | Studies/Patients | Severe Grade | I^2^ |
| PD-1/PD-L1 | | 31/2875 | 0.1156 [0.0889; 0.1490] | 80.90% | 30/2808 | 0.0115 [0.0075; 0.0176] | 0.00% | | 33/3333 | 0.1014 [0.0798; 0.1280] | 76.50% | 32/3023 | 0.011 [0.0074; 0.0162] | 0.00% |
| PD-1 | | 26/2607 | 0.1239 [0.0942; 0.1614] | 82.10% | 25/2540 | 0.0112 [0.0071; 0.0177] | 0.00% | | 25/2539 | 0.1113 [0.0870; 0.1412] | 73.40% | 25/2539 | 0.0108 [0.0070; 0.0165] | 0.00% |
| Nivolumab | | 23/2367 | 0.1307 [0.0991; 0.1705] | 81.60% | 23/2367 | 0.0117 [0.0073; 0.0186] | 0.00% | | 22/2306 | 0.1242 [0.0987; 0.1552] | 70.50% | 22/2306 | 0.0104 [0.0066; 0.0165] | 0.00% |
| 0.1 mg/kg | | 1/18 |  |  | 1/18 |  |  | | 1/18 |  |  | 1/18 |  |  |
| 0.3 mg/kg | | 3/101 | 0.1097 [0.0618; 0.1874] | 0.00% | 3/101 | 0.0164 [0.0033; 0.0775] | 0.00% | | 3/101 | 0.1295 [0.0644; 0.2432] | 33.40% | 3/101 | 0.0164 [0.0033; 0.0775] | 0.00% |
| 1 mg/kg | | 1/79 |  |  | 1/79 |  |  | | 1/79 |  |  | 1/79 |  |  |
| 2 mg/kg | | 2/76 | 0.1248 [0.0628; 0.2328] | 13.20% | 2/76 | 0.0258 [0.0065; 0.0972] | 0.00% | | 2/76 | 0.0793 [0.0360; 0.1655] | 0.00% | 2/76 | 0.0140 [0.0020; 0.0931] | 0.00% |
| 3 mg/kg | | 10/1721 | 0.1222 [0.0751; 0.1928] | 91.70% | 10/1721 | 0.0071 [0.0035; 0.0141] | 0.00% | | 9/1660 | 0.1091 [0.0765; 0.1532] | 82.60% | 9/1660 | 0.0090 [0.0046; 0.0172] | 19.30% |
| 10 mg/kg | | 2/184 | 0.0834 [0.0509; 0.1339] | 0.00% | 2/184 | 0.0108 [0.0027; 0.0422] | 0.00% | | 2/184 | 0.0996 [0.0636; 0.1527] | 0.00% | 2/184 | 0.0059 [0.0008; 0.0406] | 0.00% |
| Pembrolizumab | | 3/240 | 0.0481 [0.0053; 0.3220] | 89.50% | 2/173 | 0.0057 [0.0008; 0.0394] | 0.00% | | 3/233 | 0.0217 [0.0090; 0.0510] | 0.00% | 3/233 | 0.0137 [0.0040; 0.0462] | 0.00% |
| 2 mg/kg | | 1/89 |  |  | 1/89 |  |  | | 1/89 |  |  | 1/89 |  |  |
| 10 mg/kg | | 1/84 |  |  | 1/84 |  |  | | 2/144 | 0.0211 [0.0068; 0.0635] | 0.00% | 2/144 | 0.0172 [0.0043; 0.0661] | 0.00% |
| PD-L1 | | 5/268 | 0.0705 [0.0315; 0.1505] | 58.30% | 5/268 | 0.0139 [0.0040; 0.0472] | 0.00% | | 8/794 | 0.0543 [0.0236; 0.1200] | 81.40% | 7/484 | 0.0119 [0.0047; 0.0298] | 0.00% |
| BMS-936559 | | 4/207 | 0.0809 [0.0309; 0.1956] | 63.60% | 4/207 | 0.0160 [0.0038; 0.0642] | 3.90% | | 4/207 | 0.0834 [0.0475; 0.1426] | 15.50% | 4/207 | 0.0160 [0.0038; 0.0642] | 3.90% |
| Atezolizumab | |  |  |  |  |  |  | | 3/499 | 0.0397 [0.0052; 0.2477] | 93.00% | 2/189 | 0.0108 [0.0027; 0.0422] | 0.00% |
| 1200mg | |  |  |  |  |  |  | | 2/429 | 0.0159 [0.0031; 0.0768] |  | 1/199 |  |  |

**Table S9** Particular incidence of cutaneous irAEs in all cancer patients (continued)

| Target and Drug |  | Rash maculopapular | | | |  |  | Vitiligo | | | |  |
| --- | --- | --- | --- | --- | --- | --- | --- | --- | --- | --- | --- | --- |
|  | Studies/Patients | Any Grade | I^2^ | Studies/Patients | Severe Grade | I^2^ | Studies/Patients | Any Grade | I^2^ | Studies/Patients | Severe Grade | I^2^ |
| PD-1/PD-L1 | 14/1865 | 0.0487 [0.0176; 0.1277] | 93.90% | 14/1865 | 0.0118 [0.0049; 0.0281] | 58.90% | 14/1217 | 0.0582 [0.0336; 0.0989] | 75.30% | 13/1150 | 0.0086 [0.0040; 0.0184] | 0.00% |
| PD-1 | 13/1746 | 0.0536 [0.0186; 0.1444] | 94.20% | 13/1746 | 0.0125 [0.0050; 0.0307] | 60.50% | 10/1010 | 0.0629 [0.0333; 0.1156] | 79.70% | 9/943 | 0.0065 [0.0026; 0.0164] | 0.00% |
| Nivolumab | 10/1540 | 0.0631 [0.0181; 0.1977] | 95.40% | 10/1540 | 0.0101 [0.0030; 0.0330] | 68.50% | 7/770 | 0.0654 [0.0372; 0.1125] | 59.70% | 7/770 | 0.0068 [0.0024; 0.0192] | 0.00% |
| 0.1 mg/kg |  |  |  |  |  |  | 1/18 |  |  | 1/18 |  |  |
| 0.3 mg/kg |  |  |  |  |  |  | 1/19 |  |  | 1/19 |  |  |
| 1 mg/kg | 1/10 |  |  | 1/10 |  |  | 1/79 |  |  | 1/79 |  |  |
| 2 mg/kg |  |  |  |  |  |  |  |  |  |  |  |  |
| 3 mg/kg | 8/1423 | 0.0563 [0.0130; 0.2129] | 96.20% | 8/1423 | 0.0090 [0.0022; 0.0361] | 74.20% | 3/524 | 0.0692 [0.0372; 0.1252] | 65.20% | 3/524 | 0.0035 [0.0007; 0.0173] | 0.00% |
| 10 mg/kg |  |  |  |  |  |  | 1/130 |  |  | 1/130 |  |  |
| Pembrolizumab | 3/206 | 0.0220 [0.0077; 0.0612] | 0.00% | 3/206 | 0.0220 [0.0077; 0.0612] | 0.00% | 3/240 | 0.0490 [0.0051; 0.3427] | 90.30% | 2/173 | 0.0057 [0.0008; 0.0394] | 0.00% |
| 2 mg/kg | 1/89 |  |  | 1/89 |  |  | 1/89 |  |  | 1/89 |  |  |
| 10 mg/kg | 2/117 | 0.0190 [0.0047; 0.0727] | 0.00% | 2/117 | 0.0190 [0.0047; 0.0727] | 0.00% | 1/84 |  |  | 1/84 |  |  |
| PD-L1 | 1/119 |  |  | 1/119 |  |  | 4/207 | 0.0444 [0.0154; 0.1214] | 42.60% | 4/207 | 0.0160 [0.0038; 0.0642] | 3.90% |
| BMS-936559 |  |  |  |  |  |  | 4/207 | 0.0444 [0.0154; 0.1214] | 42.60% | 4/207 | 0.0160 [0.0038; 0.0642] | 3.90% |
| Atezolizumab | 1/119 |  |  | 1/119 |  |  |  |  |  |  |  |  |
| 1200mg |  |  |  |  |  |  |  |  |  |  |  |  |

**Table S9** Particular incidence of cutaneous irAEs in all cancer patients (continued)

| Target and Drug |  | Dermatitis | | | |  |
| --- | --- | --- | --- | --- | --- | --- |
|  | Studies/Patients | Any Grade | I^2^ | Studies/Patients | Severe Grade | I^2^ |
| PD-1/PD-L1 |  |  |  |  |  |  |
| PD-1 | 5/750 | 0.015 [0.0078; 0.0286] | 57.90% | 5/750 | 0.011 [0.0040; 0.0299] | 27.30% |
| Nivolumab | 2/555 | 0.0102 [0.0021; 0.0484] | 57.90% | 2/555 | 0.0040 [0.0010; 0.0159] | 0.00% |
| 0.1 mg/kg |  |  |  |  |  |  |
| 0.3 mg/kg |  |  |  |  |  |  |
| 1 mg/kg |  |  |  |  |  |  |
| 2 mg/kg |  |  |  |  |  |  |
| 3 mg/kg | 2/555 | 0.0102 [0.0021; 0.0484] | 57.90% | 2/555 | 0.0040 [0.0010; 0.0159] | 0.00% |
| 10 mg/kg |  |  |  |  |  |  |
| Pembrolizumab | 3/195 | 0.0170 [0.0055; 0.0513] | 0.00% | 3/195 | 0.0215 [0.0075; 0.0597] | 0.00% |
| 2 mg/kg | 1/61 |  |  | 1/61 |  |  |
| 10 mg/kg | 1/33 |  |  | 1/33 |  |  |
| PD-L1 |  |  |  |  |  |  |
| BMS-936559 |  |  |  |  |  |  |
| Atezolizumab |  |  |  |  |  |  |
| 1200mg |  |  |  |  |  |  |

**Table S10** Particular incidence of endocrinologic irAEs in all cancer patients

| Target and Drug | Hypothyroidism | | | | | | Hyperthyroidism | | | | |  |
| --- | --- | --- | --- | --- | --- | --- | --- | --- | --- | --- | --- | --- |
|  | Studies/Patients | Any Grade | I^2^ | Studies/Patients | Severe Grade | I^2^ | Studies/Patients | Any Grade | I^2^ | Studies/Patients | Severe Grade | I^2^ |
| PD-1／PD-L1 | 54/6735 | 0.0724 [0.0629; 0.0830] | 48.10% | 51/6103 | 0.0081 [0.0057; 0.0115] | 0.00% | 28/4855 | 0.0355 [0.0288; 0.0439] | 39.80% | 28/4855 | 0.0047 [0.0030; 0.0075] | 0.00% |
| PD-1 | 47/6251 | 0.0748 [0.0650; 0.0861] | 48.60% | 45/5689 | 0.0079 [0.0055; 0.0114] | 0.00% | 27/4667 | 0.0356 [0.0287; 0.0442] | 41.60% | 27/4667 | 0.0047 [0.0029; 0.0075] | 0.00% |
| Nivolumab | 27/2445 | 0.0680 [0.0534; 0.0862] | 47.20% | 27/2445 | 0.0108 [0.0067; 0.0174] | 0.00% | 13/1826 | 0.0284 [0.0207; 0.0389] | 10.60% | 13/1826 | 0.0060 [0.0030; 0.0123] | 0.00% |
| 0.3 mg/kg | 3/101 | 0.0403 [0.0152; 0.1026] | 0.00% | 3/101 | 0.0164 [0.0033; 0.0775] | 0.00% | 1/19 |  |  | 1/19 |  |  |
| 1 mg/kg | 2/89 | 0.1178 [0.0055; 0.7626] | 91.20% | 2/89 | 0.0167 [0.0023; 0.1099] | 0.00% | 1/79 |  |  | 1/79 |  |  |
| 2 mg/kg | 2/76 | 0.0671 [0.0282; 0.1514] | 0.00% | 2/76 | 0.0258 [0.0065; 0.0972] | 0.00% |  |  |  |  |  |  |
| 3 mg/kg | 12/1768 | 0.0588 [0.0420; 0.0816] | 57.50% | 12/1768 | 0.0049 [0.0022; 0.0108] | 0.00% | 8/1473 | 0.0261 [0.0174; 0.0390] | 25.20% | 8/1473 | 0.0034 [0.0013; 0.0091] | 0.00% |
| 10 mg/kg | 3/205 | 0.0444 [0.0203; 0.0944] | 18.00% | 3/205 | 0.0068 [0.0025; 0.0186] | 13.40% | 1/130 |  |  | 1/130 |  |  |
| Pembrolizumab | 20/3806 | 0.0800 [0.0677; 0.0943] | 46.40% | 18/3244 | 0.0052 [0.0030; 0.0091] | 0.00% | 14/2841 | 0.0388 [0.0293; 0.0512] | 49.70% | 14/2841 | 0.0039 [0.0021; 0.0072] | 0.00% |
| 2 mg/kg | 4/667 | 0.0691 [0.0513; 0.0926] | 4.00% | 4/667 | 0.0037 [0.0009; 0.0146] | 0.00% | 3/606 | 0.0347 [0.0225; 0.0531] | 0.00% | 3/606 | 0.0028 [0.0006; 0.0139] | 0.00% |
| 10 mg/kg | 7/1093 | 0.0762 [0.0544; 0.1056] | 42.70% | 7/1093 | 0.0068 [0.0025; 0.0186] | 13.40% | 7/1070 | 0.0354 [0.0219; 0.0568] | 39.90% | 7/1070 | 0.0048 [0.0019; 0.0121] | 0.00% |
| 200mg | 2/420 | 0.0749 [0.0529; 0.1051] | 2.9% | 2/420 | 0.0025 [0.0003; 0.0172] | 0.00% | 2/420 | 0.0548 [0.0265; 0.1099] | 67.40% | 2/420 | 0.0025 [0.0003; 0.0172] | 0.00% |
| PD-L1 | 7/484 | 0.0461 [0.0254; 0.0822] | 35.00% | 6/414 | 0.0106 [0.0034; 0.0327] | 0.00% | 1/88 |  |  | 1/88 |  |  |
| BMS-936559 | 4/207 | 0.0413 [0.0207; 0.0805] | 0.00% | 4/207 | 0.0160 [0.0038; 0.0642] | 3.90% |  |  |  |  |  |  |
| Atezolizumab | 2/189 | 0.0349 [0.0030; 0.3062] | 82.40% | 1/119 |  |  |  |  |  |  |  |  |

**Table S10** Particular incidence of endocrinologic irAEs in all cancer patients (continued)

| Target and Drug |  | Hypophysitis | | | |  |  | Thyroiditis | | | |  |
| --- | --- | --- | --- | --- | --- | --- | --- | --- | --- | --- | --- | --- |
|  | Studies/Patients | Any Grade | I^2^ | Studies/Patients | Severe Grade | I^2^ | Studies/Patients | Any Grade | I^2^ | Studies/Patients | Severe Grade | I^2^ |
| PD-1／PD-L1 |  |  |  |  |  |  | 15/2695 | 0.0162 [0.0103; 0.0253] | 35.20% | 15/2695 | 0.0043 [0.0021; 0.0088] | 0.00% |
| PD-1 | 15/3432 | 0.0085 [0.0058; 0.0126] | 0.00% | 15/3432 | 0.0059 [0.0038; 0.0093] | 0.00% | 11/2488 | 0.0143 [0.0093; 0.0221] | 24.90% | 11/2488 | 0.0027 [0.0012; 0.0062] | 0.00% |
| Nivolumab | 3/755 | 0.0054 [0.0020; 0.0143] | 0.00% | 3/755 | 0.0040 [0.0013; 0.0124] | 0.00% | 4/692 | 0.0113 [0.0041; 0.0308] | 35.30% | 4/692 | 0.0035 [0.0009; 0.0138] | 0.00% |
| 0.3 mg/kg  1 mg/kg  2 mg/kg  3 mg/kg | 3/755 | 0.0054 [0.0020; 0.0143] | 0.00% | 3/755 | 0.0040 [0.0013; 0.0124] | 0.00% | 4/692 | 0.0113 [0.0041; 0.0308] | 35.30% | 4/692 | 0.0035 [0.0009; 0.0138] | 0.00% |
| 10 mg/kg |  |  |  |  |  |  |  |  |  |  |  |  |
| Pembrolizumab | 12/2677 | 0.0089 [0.0058; 0.0138] | 0.00% | 12/2677 | 0.0059 [0.0035; 0.0099] | 0.00% | 7/1796 | 0.0142 [0.0085; 0.0236] | 24.40% | 7/1796 | 0.0023 [0.0008; 0.0066] | 0.00% |
| 2 mg/kg | 3/606 | 0.0085 [0.0024; 0.0296] | 36.550% | 3/606 | 0.0060 [0.0021; 0.0170] | 0.00% | 1/339 |  |  | 1/339 |  |  |
| 10 mg/kg | 4/883 | 0.0071 [0.0032; 0.0157] | 0.00% | 4/883 | 0.0046 [0.0017; 0.0123] | 0.00% | 2/382 | 0.0091 [0.0004; 0.1836] | 75.10% | 2/382 | 0.0043 [0.0005; 0.0343] | 13.90% |
| 200mg | 1/154 |  |  | 1/154 |  |  | 2/420 | 0.0154 [0.0046; 0.0503] | 52.00% | 2/420 | 0.0025 [0.0003; 0.0172] | 0.00% |
| PD-L1 |  |  |  |  |  |  | 4/207 | 0.0312 [0.0074; 0.1226] | 38.00% | 4/207 | 0.0160 [0.0038; 0.0642] | 3.90% |
| BMS-936559 |  |  |  |  |  |  | 4/207 | 0.0312 [0.0074; 0.1226] | 38.00% | 4/207 | 0.0160 [0.0038; 0.0642] | 3.90% |
| Atezolizumab |  |  |  |  |  |  |  |  |  |  |  |  |

**Table S10** Particular incidence of endocrinologic irAEs in all cancer patients (continued)

| Target and Drug | |  | | | Adrenal insufficiency | | |  | |
| --- | --- | --- | --- | --- | --- | --- | --- | --- | --- |
|  | Studies/Patients | | Any Grade | I^2^ | | Studies/Patients | Severe Grade | | I^2^ |
| PD-1／PD-L1 | 12/1681 | | 0.0183 [0.0102; 0.0327] | 46.70% | | 12/1681 | 0.0117 [0.0062; 0.0219] | | 21.80% |
| PD-1 | 8/1474 | | 0.0130 [0.0057; 0.0292] | 57.70% | | 8/1474 | 0.0093 [0.0046; 0.0188] | | 19.50% |
| Nivolumab | 4/466 | | 0.0174 [0.0044; 0.0663] | 63.40% | | 4/466 | 0.0160 [0.0064; 0.0390] | | 18.50% |
| 0.3 mg/kg  1 mg/kg  2 mg/kg  3 mg/kg | 4/466 | | 0.0174 [0.0044; 0.0663] | 63.40% | | 4/466 | 0.0160 [0.0064; 0.0390] | | 18.50% |
| 10 mg/kg  Pembrolizumab | 4/1008 | | 0.0098 [0.0041; 0.0231] | 33.30% | | 4/1008 | 0.0045 [0.0017; 0.0120] | | 0.00% |
| 2 mg/kg | 1/339 | |  |  | | 1/339 |  | |  |
| 10 mg/kg | 2/403 | | 0.0161 [0.0043; 0.0581] | 54.10% | | 2/403 | 0.0051 [0.0013; 0.0202] | | 0.00% |
| 200mg | 1/266 | |  |  | | 1/266 |  | |  |
| PD-L1 | 4/207 | | 0.0238 [0.0089; 0.0620] | 0.00% | | 4/207 | 0.0244 [0.0070; 0.0811] | | 11.70% |
| BMS-936559 | 4/207 | | 0.0238 [0.0089; 0.0620] | 0.00% | | 4/207 | 0.0244 [0.0070; 0.0811] | | 11.70% |

**Table S11** Particular incidence of gastrointestinal irAEs in all cancer patients

| Target and Drug | Colitis | | | | | | Diarrhea | | | | | |
| --- | --- | --- | --- | --- | --- | --- | --- | --- | --- | --- | --- | --- |
|  | Studies/Patients | Any Grade | I^2^ | Studies/Patients | Severe Grade | I^2^ | Studies/Patients | Any Grade | I^2^ | Studies/Patients | Severe Grade | I^2^ |
| PD-1/PD-L1 | 31/5421 | 0.0207 [0.0158; 0.0272] | 38.30% | 30/5354 | 0.0156 [0.0114; 0.0214] | 32.90% | 33/3082 | 0.1128 [0.0904; 0.1399] | 72.30% | 32/3015 | 0.0175 [0.0128; 0.0238] | 0.00% |
| PD-1 | 28/4551 | 0.0224 [0.0170; 0.0293] | 33.80% | 27/4484 | 0.0165 [0.0120; 0.0228] | 32.00% | 26/2607 | 0.1217 [0.0965; 0.1524] | 73.30% | 25/2540 | 0.0183 [0.0132; 0.0254] | 0.00% |
| Nivolumab | 12/1470 | 0.0250 [0.0133; 0.0466] | 56.40% | 12/1470 | 0.0194 [0.0093; 0.0401] | 56.70% | 23/2367 | 0.1338 [0.1069; 0.1662] | 71.60% | 23/2367 | 0.0189 [0.0136; 0.0263] | 0.00% |
| 0.1 mg/kg |  |  |  |  |  |  | 1/18 |  |  | 1/18 |  |  |
| 0.3 mg/kg | 1/22 |  |  | 1/22 |  |  | 3/101 | 0.0935 [0.0332; 0.2365] | 52.60% | 3/101 | 0.0164 [0.0033; 0.0775] | 0.00% |
| 1 mg/kg | 1/6 |  |  | 1/6 |  |  | 1/79 |  |  | 1/79 |  |  |
| 2 mg/kg | 1/22 |  |  | 1/22 |  |  | 2/76 | 0.1346 [0.0739; 0.2328] | 0.00% | 2/76 | 0.0140 [0.0020; 0.0931] | 0.00% |
| 3 mg/kg | 6/1266 | 0.0120 [0.0072; 0.0198] | 0.00% | 6/1266 | 0.0081 [0.0044; 0.0150] | 0.00% | 10/1721 | 0.1175 [0.0831; 0.1634] | 83.20% | 10/1721 | 0.0155 [0.0099; 0.0241] | 4.20% |
| 10 mg/kg |  |  |  |  |  |  | 2/184 | 0.1122 [0.0701; 0.1747] | 17.1% | 2/184 | 0.0233 [0.0088; 0.0605] | 0.00% |
| 10 mg/kg naïve | 1/24 |  |  | 1/24 |  |  | 1/24 |  |  | 1/24 |  |  |
| 10 mg/kg treated | 1/23 |  |  | 1/23 |  |  | 1/23 |  |  | 1/23 |  |  |
| Pembrolizumab | 16/3081 | 0.0202 [0.0154; 0.0263] | 1.40% | 15/3014 | 0.0161 [0.0119; 0.0218] | 0.00% | 3/240 | 0.0397 [0.0168; 0.0908] | 37.20% | 2/173 | 0.0057 [0.0008; 0.0394] | 0.00% |
| 2 mg/kg | 3/578 | 0.0189 [0.0072; 0.0489] | 52.80% | 3/578 | 0.0144 [0.0040; 0.0502] | 54.50% | 1/89 |  |  | 1/89 |  |  |
| 10 mg/kg | 6/982 | 0.0196 [0.0103; 0.0368] | 30.70% | 6/982 | 0.0181 [0.0101; 0.0322] | 14.50% | 1/84 |  |  | 1/84 |  |  |
| 200mg | 2/420 | 0.0215 [0.0112; 0.0408] | 0.00% | 2/420 | 0.0119 [0.0050; 0.0283] | 0.00% |  |  |  |  |  |  |
| PD-L1 | 3/870 | 0.0071 [0.0028; 0.0176] | 7.80% | 3/870 | 0.0070 [0.0018; 0.0262] | 33.80% | 7/475 | 0.0740 [0.0384; 0.1377] | 59.20% | 7/475 | 0.0121 [0.0048; 0.0302] | 0.00% |
| BMS-936559 |  |  |  |  |  |  | 4/407 | 0.1040 [0.0602; 0.1738] | 27.60% | 4/407 | 0.0160 [0.0038; 0.0642] | 3.90% |
| 0.3 mg/kg |  |  |  |  |  |  | 1/3 |  |  | 1/3 |  |  |
| 1 mg/kg |  |  |  |  |  |  | 1/37 |  |  | 1/37 |  |  |
| 3 mg/kg |  |  |  |  |  |  | 1/42 |  |  | 1/42 |  |  |
| 10 mg/kg |  |  |  |  |  |  | 1/125 |  |  | 1/125 |  |  |
| Durvalumab |  |  |  |  |  |  | 1/61 |  |  | 1/61 |  |  |
| 10 mg/kg |  |  |  |  |  |  | 1/61 |  |  | 1/61 |  |  |
| Atezolizumab | 3/870 | 0.0071 [0.0028; 0.0176] | 7.80% | 3/870 | 0.0070 [0.0018; 0.0262] | 33.80% | 1/119 |  |  | 1/119 |  |  |
| 1200mg | 3/870 | 0.0071 [0.0028; 0.0176] | 7.80% | 3/870 | 0.0070 [0.0018; 0.0262] | 33.80% | 1/119 |  |  | 1/119 |  |  |

**Table S11** Particular incidence of gastrointestinal irAEs in all cancer patients (continued)

| Target and Drug | | Pancreatitis | | | | | |
| --- | --- | --- | --- | --- | --- | --- | --- |
|  | Studies/Patients | | Any Grade | I^2^ | Studies/Patients | Severe Grade | I^2^ |
| PD-1/PD-L1 | 7/1664 | | 0.0077 [0.0041; 0.0144] | 0.00% | 7/1664 | 0.0067 [0.0034; 0.0131] | 0.00% |
| PD-1 | 7/1664 | | 0.0077 [0.0041; 0.0144] | 0.00% | 7/1664 | 0.0067 [0.0034; 0.0131] | 0.00% |
| Nivolumab |  | |  |  |  |  |  |
| Pembrolizumab | 7/1664 | | 0.0077 [0.0041; 0.0144] | 0.00% | 7/1664 | 0.0067 [0.0034; 0.0131] | 0.00% |
| 2 mg/kg | 2/428 | | 0.0095 [0.0036; 0.0251] | 0.00% | 2/428 | 0.0070 [0.0023; 0.0215] | 0.00% |
| 10 mg/kg | 2/427 | | 0.0066 [0.0006; 0.0689] | 57.50% | 2/427 | 0.0066 [0.0006; 0.0689] | 57.50% |
| 200mg | 1/54 | |  |  | 1/54 |  |  |
| PD-L1 |  | |  |  |  |  |  |
| BMS-936559 |  | |  |  |  |  |  |
| Durvalumab |  | |  |  |  |  |  |
| Atezolizumab |  | |  |  |  |  |  |

**Table S12** Particular incidence of hepatic irAEs in all cancer patients

| Target and Drug | | AST increased | | | | | | ALT increased | | | | | | |
| --- | --- | --- | --- | --- | --- | --- | --- | --- | --- | --- | --- | --- | --- | --- |
|  | Studies/Patients | | Any Grade | I^2^ | Studies/Patients | Severe Grade | I^2^ | | Studies/Patients | Any Grade | I^2^ | Studies/Patients | Severe Grade | I^2^ |
| PD-1／PD-L1 | 29/2990 | | 0.0439 [0.0310; 0.0618] | 63.60% | 28/2680 | 0.0181 [0.0123; 0.0266] | 17.10% | | 36/3436 | 0.0416 [0.0313; 0.0551] | 51.00% | 35/3126 | 0.0194 [0.0143; 0.0263] | 2.10% |
| PD-1 | 26/2419 | | 0.0486 [0.0338; 0.0694] | 62.50% | 26/2419 | 0.0177 [0.0115; 0.0274] | 22.00% | | 28/2570 | 0.0478 [0.0350; 0.0650] | 52.60% | 28/2570 | 0.0192 [0.0131; 0.0280] | 13.70% |
| Nivolumab | 23/2236 | | 0.0503 [0.0344; 0.0731] | 64.40% | 23/2236 | 0.0169 [0.0111; 0.0257] | 10.80% | | 25/2387 | 0.0491 [0.0357; 0.0671] | 53.30% | 25/2387 | 0.0182 [0.0128; 0.0260] | 0.20% |
| 0.1 mg/kg | 1/18 | |  |  | 1/18 |  |  | |  |  |  |  |  |  |
| 0.3 mg/kg | 3/101 | | 0.0403 [0.0152; 0.1026] | 0.00% | 3/101 | 0.0372 [0.0131; 0.1014] | 0.00% | | 3/101 | 0.0542 [0.0227; 0.1240] | 0.00% | 3/101 | 0.0372 [0.0131; 0.1014] | 0.00% |
| 1 mg/kg | 2/89 | | 0.1665 [0.0037; 0.9141] | 94.30% | 2/89 | 0.0167 [0.0023; 0.1099] | 0.00% | | 2/89 | 0.1546 [0.0152; 0.6848] | 89.30% | 2/89 | 0.0258 [0.0065; 0.0972] | 0.00% |
| 2 mg/kg | 2/76 | | 0.0714 [0.0300; 0.1607] | 0.00% | 2/76 | 0.0258 [0.0065; 0.0972] | 0.00% | | 2/76 | 0.0401 [0.0130; 0.1173] | 0.00% | 2/76 | 0.0258 [0.0065; 0.0972] | 0.00% |
| 3 mg/kg | 10/1614 | | 0.0319 [0.0207; 0.0487] | 44.20% | 10/1614 | 0.0104 [0.0061; 0.0179] | 0.00% | | 11/1731 | 0.0284 [0.0199; 0.0403] | 21.20% | 11/1731 | 0.0111 [0.0067; 0.0182] | 0.00% |
| 10 mg/kg | 1/54 | |  |  | 1/54 |  |  | | 2/184 | 0.0396 [0.0190; 0.0807] | 0.00% | 2/184 | 0.0169 [0.0054; 0.0510] | 0.00% |
| 10 mg/kg naive | 1/24 | |  |  | 1/24 |  |  | |  |  |  |  |  |  |
| 10 mg/kg treated | 1/23 | |  |  | 1/23 |  |  | |  |  |  |  |  |  |
| Pembrolizumab | 3/183 | | 0.0235 [0.0056; 0.0928] | 36.20% | 3/183 | 0.0210 [0.0019; 0.1968] | 67.60% | | 3/183 | 0.0291 [0.0047; 0.1599] | 62.20% | 3/183 | 0.0210 [0.0019; 0.1968] | 67.60% |
| 2 mg/kg | 1/89 | |  |  | 1/89 |  |  | | 1/89 |  |  | 1/89 |  |  |
| 10 mg/kg | 1/84 | |  |  | 1/84 |  |  | | 1/84 |  |  | 1/84 |  |  |
| PD-L1 | 3/571 | | 0.0153 [0.0038; 0.0595] | 69.70% | 2/261 | 0.0168 [0.0063; 0.0438] | 0.00% | | 8/866 | 0.0232 [0.0132; 0.0404] | 20.00% | 7/556 | 0.0202 [0.0107; 0.0378] | 0.00% |
| BMS-936559 |  | |  |  |  |  |  | | 4/407 | 0.0245 [0.0092; 0.0639] | 0.00% | 4/407 | 0.0160 [0.0038; 0.0642] | 3.90% |
| 0.3 mg/kg |  | |  |  |  |  |  | | 1/3 |  |  | 1/3 |  |  |
| 1 mg/kg |  | |  |  |  |  |  | | 1/37 |  |  | 1/37 |  |  |
| 3 mg/kg |  | |  |  |  |  |  | | 1/42 |  |  | 1/42 |  |  |
| 10 mg/kg |  | |  |  |  |  |  | | 1/125 |  |  | 1/125 |  |  |
| Atezolizumab | 3/571 | | 0.0153 [0.0038; 0.0595] | 69.70% | 2/261 | 0.0168 [0.0063; 0.0438] | 0.00% | | 3/571 | 0.0186 [0.0059; 0.0570] | 65.20% | 2/261 | 0.0193 [0.0080; 0.0455] | 0.00% |
| 1200mg | 3/571 | | 0.0153 [0.0038; 0.0595] | 69.70% | 2/261 | 0.0168 [0.0063; 0.0438] | 0.00% | | 3/571 | 0.0186 [0.0059; 0.0570] | 65.20% | 2/261 | 0.0193 [0.0080; 0.0455] | 0.00% |
| Avelumab |  | |  |  |  |  |  | | 1/88 |  |  |  |  |  |

**Table S12** Particular incidence of hepatic irAEs in all cancer patients (continued)

| Target and Drug | Blood bilirubin increased | | | | | | | Hepatitis | | | | | | |
| --- | --- | --- | --- | --- | --- | --- | --- | --- | --- | --- | --- | --- | --- | --- |
|  | | Studies/Patients | Any Grade | I^2^ | Studies/Patients | Severe Grade | I^2^ | | Studies/Patients | Any Grade | I^2^ | Studies/Patients | Severe Grade | I^2^ |
| PD-1／PD-L1 | | 9/1070 | 0.0201 [0.0085; 0.0469] | 59.60% | 9/1070 | 0.0126 [0.0053; 0.0297] | 23.50% | | 12/2641 | 0.0122 [0.0084; 0.0176] | 0.00% | 12/2641 | 0.01 [0.0066; 0.0151] | 0.00% |
| PD-1 | | 8/851 | 0.0196 [0.0070; 0.0533] | 64.60% | 8/851 | 0.0101 [0.0034; 0.0294] | 29.60% | | 10/1890 | 0.0138 [0.0093; 0.0205] | 0.00% | 10/1890 | 0.0115 [0.0074; 0.0180] | 0.00% |
| Nivolumab | | 8/851 | 0.0196 [0.0070; 0.0533] | 64.60% | 8/851 | 0.0101 [0.0034; 0.0294] | 29.60% | |  |  |  |  |  |  |
| 0.1 mg/kg | |  |  |  |  |  |  | |  |  |  |  |  |  |
| 0.3 mg/kg | | 1/22 |  |  | 1/22 |  |  | |  |  |  |  |  |  |
| 1 mg/kg | |  |  |  |  |  |  | |  |  |  |  |  |  |
| 2 mg/kg | | 1/22 |  |  | 1/22 |  |  | |  |  |  |  |  |  |
| 3 mg/kg | | 4/860 | 0.0076 [0.0034; 0.0167] | 0.00% | 4/860 | 0.0024 [0.0006; 0.0096] | 0.00% | |  |  |  |  |  |  |
| 10 mg/kg | |  |  |  |  |  |  | |  |  |  |  |  |  |
| 10 mg/kg naïve | | 1/24 |  |  | 1/24 |  |  | |  |  |  |  |  |  |
| 10 mg/kg treated | | 1/23 |  |  | 1/23 |  |  | |  |  |  |  |  |  |
| Pembrolizumab | |  |  |  |  |  |  | | 10/1890 | 0.0138 [0.0093; 0.0205] | 0.00% | 10/1890 | 0.0115 [0.0074; 0.0180] | 0.00% |
| 2 mg/kg | |  |  |  |  |  |  | | 2/267 | 0.0112 [0.0036; 0.0342] | 0.00% | 2/267 | 0.0076 [0.0019; 0.0297] | 0.00% |
| 10 mg/kg | |  |  |  |  |  |  | | 5/690 | 0.0179 [0.0101; 0.0317] | 0.00% | 5/690 | 0.0159 [0.0087; 0.0288] | 0.00% |
| PD-L1 | | 1/119 | 0.0168 [0.0042; 0.0647] |  | 1/119 | 0.0168 [0.0042; 0.0647] |  | | 2/751 | 0.0042 [0.0014; 0.0130] | 0.00% | 2/751 | 0.0040 [0.0013; 0.0123] | 0.00% |
| BMS-936559 | |  |  |  |  |  |  | |  |  |  |  |  |  |
| Atezolizumab | | 1/119 |  |  | 1/119 |  |  | | 2/751 | 0.0042 [0.0014; 0.0130] | 0.00% | 2/751 | 0.0040 [0.0013; 0.0123] | 0.00% |
| Avelumab | |  |  |  |  |  |  | |  |  |  |  |  |  |

**Table S13** Particular incidence of pulmonary irAEs in all cancer patients

| Target and Drug | Pneumonitis | | | | | | Lung infiltration | | | | | |
| --- | --- | --- | --- | --- | --- | --- | --- | --- | --- | --- | --- | --- |
|  | Studies/Patients | Any Grade | I^2^ | Studies/Patients | Severe Grade | I^2^ | Studies/Patients | Any Grade | I^2^ | Studies/Patients | Severe Grade | I^2^ |
| PD-1／PD-L1 | 44/7060 | 0.033 [0.0275; 0.0395] | 35.00% | 43/6750 | 0.0172 [0.0139; 0.0214] | 2.80% | 2/238 | 0.0139 [0.0045; 0.0421] | 0.00% | 2/238 | 0.0042 [0.0006; 0.0291] | 0.00% |
| PD-1 | 40/5911 | 0.0360 [0.0304; 0.0426] | 21.60% | 40/5911 | 0.0186 [0.0149; 0.0230] | 0.00% | 2/238 | 0.0139 [0.0045; 0.0421] | 0.00% | 2/238 | 0.0042 [0.0006; 0.0291] | 0.00% |
| Nivolumab | 22/2333 | 0.0375 [0.0290; 0.0484] | 18.90% | 22/2333 | 0.0185 [0.0130; 0.0264] | 0.00% | 2/238 | 0.0139 [0.0045; 0.0421] | 0.00% | 2/238 | 0.0042 [0.0006; 0.0291] | 0.00% |
| 0.1 mg/kg | 1/18 |  |  | 1/18 |  |  |  |  |  |  |  |  |
| 0.3 mg/kg | 3/101 | 0.0550 [0.0240; 0.1210] | 0.00% | 3/101 | 0.0352 [0.0102; 0.1149] | 0.00% |  |  |  |  |  |  |
| 1 mg/kg | 1/79 |  |  | 1/79 |  |  |  |  |  |  |  |  |
| 2 mg/kg | 2/76 | 0.0401 [0.0130; 0.1173] | 0.00% | 2/76 | 0.0140 [0.0020; 0.0931] | 0.00% |  |  |  |  |  |  |
| 3 mg/kg | 10/1721 | 0.0289 [0.0209; 0.0398] | 16.20% | 10/1721 | 0.0125 [0.0077; 0.0203] | 0.00% | 1/131 |  |  | 1/131 |  |  |
| 10 mg/kg | 2/184 | 0.0441 [0.0222; 0.0857] | 0.00% | 2/184 | 0.0300 [0.0125; 0.0702] | 0.00% |  |  |  |  |  |  |
| 10 mg/kg naïve | 1/24 |  |  | 1/24 |  |  |  |  |  |  |  |  |
| 10 mg/kg treated | 1/23 |  |  | 1/23 |  |  |  |  |  |  |  |  |
| Pembrolizumab | 18/3578 | 0.0323 [0.0253; 0.0412] | 28.30% | 18/3578 | 0.0182 [0.0135; 0.0244] | 7.80% |  |  |  |  |  |  |
| 2 mg/kg | 4/667 | 0.0309 [0.0164; 0.0575] | 31.90% | 4/667 |  |  |  |  |  |  |  |  |
| 10 mg/kg | 5/922 | 0.0263 [0.0155; 0.0444] | 26.90% | 5/922 |  |  |  |  |  |  |  |  |
| 200mg | 2/420 | 0.0483 [0.0314; 0.0737] | 0.00% | 2/420 |  |  |  |  |  |  |  |  |
| PD-L1 | 4/1149 | 0.0128 [0.0067; 0.0244] | 23.00% | 3/839 | 0.0078 [0.0036; 0.0168] | 0.00% |  |  |  |  |  |  |
| Atezolizumab | 3/1061 | 0.0129 [0.0057; 0.0290] | 48.40% | 2/751 | 0.0067 [0.0028; 0.0159] | 0.00% |  |  |  |  |  |  |
| Avelumab | 1/88 |  |  | 1/88 |  |  |  |  |  |  |  |  |

**Table S13** Particular incidence of pulmonary irAEs in all cancer patients (continued)

| Target and Drug | Interstitial lung disease | | | | | |
| --- | --- | --- | --- | --- | --- | --- |
|  | Studies/Patients | Any Grade | I^2^ | Studies/Patients | Severe Grade | I^2^ |
| PD-1／PD-L1 |  |  |  |  |  |  |
| PD-1 | 2/326 | 0.011 [0.0032; 0.0370] | 12.40% | 2/326 | 0.0065 [0.0016; 0.0255] | 0.00% |
| Nivolumab | 1/287 |  |  | 1/287 |  |  |
| 0.1 mg/kg  0.3 mg/kg  1 mg/kg  2 mg/kg  3 mg/kg | 1/287 |  |  | 1/287 |  |  |
| 10 mg/kg |  |  |  |  |  |  |
| 10 mg/kg naïve |  |  |  |  |  |  |
| 10 mg/kg treated |  |  |  |  |  |  |
| Pembrolizumab | 1/39 |  |  | 1/39 |  |  |
| 2 mg/kg  10 mg/kg | 1/39 |  |  | 1/39 |  |  |
| PD-L1 |  |  |  |  |  |  |
| Atezolizumab |  |  |  |  |  |  |
| Avelumab |  |  |  |  |  |  |

**Table S14** Particular incidence of renal irAEs in all cancer patients

| Target and Drug | Blood creatinine increased | | | | | | Nephritis | | | | | |
| --- | --- | --- | --- | --- | --- | --- | --- | --- | --- | --- | --- | --- |
|  | Studies/Patients | Any Grade | I^2^ | Studies/Patients | Severe Grade | I^2^ | Studies/Patients | Any Grade | I^2^ | Studies/Patients | Severe Grade | I^2^ |
| PD-1／PD-L1 | 13/1329 | 0.0379 [0.0211; 0.0670] | 65.60% | 13/1329 | 0.0072 [0.0033; 0.0154] | 0.00% | 8/1551 | 0.0088 [0.0050; 0.0154] | 0.00% | 8/1551 | 0.0066 [0.0033; 0.0132] | 0.00% |
| PD-1 | 13/1329 | 0.0379 [0.0211; 0.0670] | 65.60% | 13/1329 | 0.0072 [0.0033; 0.0154] | 0.00% | 7/1463 | 0.0067 [0.0033; 0.0136] | 0.00% | 7/1463 | 0.0067 [0.0033; 0.0136] | 0.00% |
| Nivolumab | 13/1329 | 0.0379 [0.0211; 0.0670] | 65.60% | 13/1329 | 0.0072 [0.0033; 0.0154] | 0.00% | 1/131 |  |  | 1/131 |  |  |
| 0.3 mg/kg | 2/82 | 0.0449 [0.0083; 0.2078] | 50.30% | 2/82 | 0.0133 [0.0019; 0.0887] | 0.00% |  |  |  |  |  |  |
| 2 mg/kg | 2/76 | 0.0580 [0.0026; 0.5945] | 78.60% | 2/76 | 0.0140 [0.0020; 0.0931] | 0.00% |  |  |  |  |  |  |
| 3 mg/kg | 6/1070 | 0.0191 [0.0105; 0.0343] | 32.10% | 6/1070 | 0.0032 [0.0010; 0.0098] | 0.00% | 1/131 |  |  | 1/131 |  |  |
| 10 mg/kg | 1/54 |  |  | 1/54 |  |  |  |  |  |  |  |  |
| 10 mg/kg naïve | 1/24 |  |  | 1/24 |  |  |  |  |  |  |  |  |
| 10 mg/kg treated | 1/23 |  |  | 1/23 |  |  |  |  |  |  |  |  |
| Pembrolizumab | |  |  |  |  |  | 6/1332 | 0.0075 [0.0039; 0.0144] | 0.00% | 6/1332 | 0.0059 [0.0026; 0.0130] | 0.00% |
| 2 mg/kg |  |  |  |  |  |  | 1/178 |  |  | 1/178 |  |  |
| 10 mg/kg |  |  |  |  |  |  | 2/456 | 0.0045 [0.0011; 0.0178] | 0.00% | 2/456 | 0.0022 [0.0003; 0.0157] | 0.00% |
| 200mg |  |  |  |  |  |  | 2/420 | 0.0072 [0.0023; 0.0220] | 0.00% | 2/420 | 0.0072 [0.0023; 0.0220] | 0.00% |
| PD-L1 |  |  |  |  |  |  | 1/88 |  |  | 1/88 |  |  |
| Avelumab |  |  |  |  |  |  | 1/88 |  |  | 1/88 |  |  |

**Table S14** Particular incidence of renal irAEs in all cancer patients (continued)

| Target and Drug | Renal failure acute | | | | | | Renal failure | | | | | |
| --- | --- | --- | --- | --- | --- | --- | --- | --- | --- | --- | --- | --- |
|  | Studies/Patients | Any Grade | I^2^ | Studies/Patients | Severe Grade | I^2^ | Studies/Patients | Any Grade | I^2^ | Studies/Patients | Severe Grade | I^2^ |
| PD-1／PD-L1 | 7/701 | 0.0251 [0.0095; 0.0650] | 59.20% | 7/701 | 0.0251 [0.0095; 0.0650] | 59.20% | 4/717 | 0.01 [0.0045; 0.0220] | 0.00% | 4/717 | 0.01 [0.0045; 0.0220] | 0.00% |
| PD-1 | 7/701 | 0.0251 [0.0095; 0.0650] | 59.20% | 7/701 | 0.0173 [0.0061; 0.0480] | 39.50% | 4/717 | 0.01 [0.0045; 0.0220] | 0.00% | 4/717 | 0.01 [0.0045; 0.0220] | 0.00% |
| Nivolumab | 7/701 | 0.0251 [0.0095; 0.0650] | 59.20% | 7/701 | 0.0251 [0.0095; 0.0650] | 59.20% | 4/717 | 0.01 [0.0045; 0.0220] | 0.00% | 4/717 | 0.01 [0.0045; 0.0220] | 0.00% |
| 0.3 mg/kg | 1/22 |  |  | 1/22 |  |  |  |  |  |  |  |  |
| 2 mg/kg | 1/22 |  |  | 1/22 |  |  |  |  |  |  |  |  |
| 3 mg/kg | 3/610 | 0.0052 [0.0017; 0.0161] | 0.00% | 3/610 | 0.0049 [0.0014; 0.0167] | 0.00% | 3/610 | 0.0073 [0.0027; 0.0192] | 0.00% | 3/610 | 0.0026 [0.0005; 0.0128] | 0.00% |
| 10 mg/kg |  |  |  |  |  |  |  |  |  |  |  |  |
| 10 mg/kg naïve | 1/24 |  |  | 1/24 |  |  |  |  |  |  |  |  |
| 10 mg/kg treated | 1/23 |  |  | 1/23 |  |  |  |  |  |  |  |  |
| Pembrolizumab |  |  |  |  |  |  |  |  |  |  |  |  |
| 2 mg/kg |  |  |  |  |  |  |  |  |  |  |  |  |
| 10 mg/kg |  |  |  |  |  |  |  |  |  |  |  |  |
| 200mg |  |  |  |  |  |  |  |  |  |  |  |  |

1. **Incidence of irAEs in melanomas patients**

**Table S15** Particular incidence of cutaneous irAEs in melanomas patients

| Target and Drug | Pruritus | | | | | | rash | | | | |  |
| --- | --- | --- | --- | --- | --- | --- | --- | --- | --- | --- | --- | --- |
|  | Studies/Patients | Any Grade | I^2^ | Studies/Patients | Severe Grade | I^2^ | Studies/Patients | Any Grade | I^2^ | Studies/Patients | Severe Grade | I^2^ |
| PD-1 | 8/1195 | 0.1653 [0.1018; 0.2572] | 90.00% | 7/1128 | 0.0071 [0.0031; 0.0163] | 0.00% | 6/1067 | 0.1181 [0.0713; 0.1895] | 86.60% | 6/1067 | 0.0057 [0.0025; 0.0125] | 0.00% |
| Nivolumab | 5/955 | 0.2191 [0.1330; 0.3392] | 91.90% | 5/955 | 0.0072 [0.0027; 0.0190] | 8.50% | 4/894 | 0.1662 [0.1111; 0.2412] | 84.30% | 4/894 | 0.0057 [0.0024; 0.0135] | 0.00% |
| 3 mg/kg | 4/848 | 0.2448 [0.1389; 0.3944] | 93.50% | 4/848 | 0.0070 [0.0021; 0.0232] | 29.30% | 3/787 | 0.1485 [0.0894; 0.2367] | 87.60% | 3/787 | 0.0039 [0.0012; 0.0119] | 0.00% |
| Pembrolizumab | 3/240 | 0.0481 [0.0053; 0.3220] | 89.50% | 2/173 | 0.0057 [0.0008; 0.0394] | 0.00% | 2/173 | 0.0231 [0.0087; 0.0600] | 0.00% | 2/173 | 0.0057 [0.0008; 0.0394] | 0.00% |

**Table S15** Particular incidence of cutaneous irAEs in melanomas patients (continued)

| Target and Drug |  | Rash maculopapular | | | |  |  | Vitiligo | | | |  |
| --- | --- | --- | --- | --- | --- | --- | --- | --- | --- | --- | --- | --- |
|  | Studies/Patients | Any Grade | I^2^ | Studies/Patients | Severe Grade | I^2^ | Studies/Patients | Any Grade | I^2^ | Studies/Patients | Severe Grade | I^2^ |
| PD-1 | 6/922 | 0.0605 [0.0101; 0.2901] | 96.80% | 6/922 | 0.0107 [0.0028; 0.0397] | 67.60% | 5/714 | 0.0693 [0.0290; 0.1569] | 87.60% | 4/647 | 0.0035 [0.0009; 0.0138] | 0.00% |
| Nivolumab | 4/749 | 0.1136 [0.0131; 0.5521] | 97.90% | 4/749 | 0.0093 [0.0013; 0.0652] | 79.00% | 2/474 | 0.0762 [0.0372; 0.1497] | 78.90% | 2/474 | 0.0021 [0.0003; 0.0149] | 0.00% |
| 3 mg/kg | 3/642 | 0.1708 [0.0135; 0.7564] | 98.50% | 3/642 | 0.0109 [0.0010; 0.1080] | 84.30% | 2/474 | 0.0762 [0.0372; 0.1497] | 78.90% | 2/474 | 0.0021 [0.0003; 0.0149] | 0.00% |
| Pembrolizumab | 2/173 | 0.0132 [0.0033; 0.0513] | 0.00% | 2/173 | 0.0132 [0.0033; 0.0513] | 0.00% | 3/240 | 0.0490 [0.0051; 0.3427] | 90.30% | 2/173 | 0.0057 [0.0008; 0.0394] | 0.00% |

**Table S16** Particular incidence of endocrinologic irAEs in melanomas patients

| Target and Drug | Hypothyroidism | | | | | | Hyperthyroidism | | | | | |
| --- | --- | --- | --- | --- | --- | --- | --- | --- | --- | --- | --- | --- |
|  | Studies/Patients | Any Grade | I^2^ | Studies/Patients | Severe Grade | I^2^ | Studies/Patients | Any Grade | I^2^ | Studies/Patients | Severe Grade | I^2^ |
| PD-1 | 14/2762 | 0.0742 [0.0606; 0.0906] | 49.80% | 13/2695 | 0.0044 [0.0023; 0.0083] | 0.00% | 13/2695 | 0.0319 [0.0241; 0.0421] | 35.00% | 13/2695 | 0.0035 [0.0018; 0.0069] | 0.00% |
| Nivolumab | 5/955 | 0.0660 [0.0506; 0.0858] | 12.00% | 5/955 | 0.0055 [0.0019; 0.0156] | 0.00% | 5/955 | 0.0334 [0.0234; 0.0474] | 0.00% | 5/955 | 0.0031 [0.0009; 0.0105] | 0.00% |
| 3 mg/kg | 4/848 | 0.0663 [0.0479; 0.0911] | 30.50% | 4/848 | 0.0028 [0.0007; 0.0109] | 0.00% | 4/848 | 0.0348 [0.0241; 0.0500] | 0.00% | 4/848 | 0.0028 [0.0007; 0.0109] | 0.00% |
| Pembrolizumab | 9/1807 | 0.0793 [0.0606; 0.1031] | 57.80% | 8/1740 | 0.0039 [0.0017; 0.0086] | 0.00% | 8/1740 | 0.0301 [0.0197; 0.0458] | 52.80% | 8/1740 | 0.0037 [0.0017; 0.0083] | 0.00% |
| 2 mg/kg | 2/267 | 0.0542 [0.0323; 0.0894] | 0.00% | 2/267 | 0.0039 [0.0006; 0.0274] | 0.00% | 2/267 | 0.0293 [0.0103; 0.0808] | 0.00% | 2/267 | 0.0039 [0.0006; 0.0274] | 0.00% |
| 10 mg/kg | 3/540 | 0.0748 [0.0445; 0.1229] | 50.60% | 3/540 | 0.0031 [0.0006; 0.0151] | 0.00% | 3/540 | 0.0262 [0.0153; 0.0447] | 0.00% | 3/540 | 0.0031 [0.0006; 0.0151] | 0.00% |

**Table S16** Particular incidence of endocrinologic irAEs in melanomas patients (continued)

| Target and Drug |  | Hypophysitis | | | |  |  | Thyroiditis | | | |  |
| --- | --- | --- | --- | --- | --- | --- | --- | --- | --- | --- | --- | --- |
|  | Studies/Patients | Any Grade | I^2^ | Studies/Patients | Severe Grade | I^2^ | Studies/Patients | Any Grade | I^2^ | Studies/Patients | Severe Grade | I^2^ |
| PD-1 | 10/2259 | 0.0093 [0.0058; 0.0147] | 0.00% | 10/2259 | 0.0053 [0.0029; 0.0095] | 0.00% | 2/655 | 0.0122 [0.0061; 0.0243] | 0.00% | 2/655 | 0.0015 [0.0002; 0.0107] | 0.00% |
| Nivolumab | 2/519 | 0.0058 [0.0019; 0.0179] | 0.00% | 2/519 | 0.0039 [0.0010; 0.0156] | 0.00% |  |  |  |  |  |  |
| 3 mg/kg | 2/519 | 0.0058 [0.0019; 0.0179] | 0.00% | 2/519 | 0.0039 [0.0010; 0.0156] | 0.00% |  |  |  |  |  |  |
| Pembrolizumab | 8/1740 | 0.0098 [0.0058; 0.0164] | 0.00% | 8/1740 | 0.0050 [0.0025; 0.0100] | 0.00% | 2/655 | 0.0122 [0.0061; 0.0243] | 0.00% | 2/655 | 0.0015 [0.0002; 0.0107] | 0.00% |
| 2 mg/kg | 2/267 | 0.0134 [0.0036; 0.0489] | 0.00% | 2/267 | 0.0076 [0.0019; 0.0297] | 0.00% |  |  |  |  |  |  |
| 10 mg/kg | 3/540 | 0.0084 [0.0033; 0.0209] | 0.00% | 3/540 | 0.0048 [0.0014; 0.0165] | 0.00% |  |  |  |  |  |  |

**Table S17** Particular incidence of gastrointestinal irAEs in melanomas patients

| Target and Drug | Colitis | | | | | | Diarrhea | | | | | |
| --- | --- | --- | --- | --- | --- | --- | --- | --- | --- | --- | --- | --- |
|  | Studies/Patients | Any Grade | I^2^ | Studies/Patients | Severe Grade | I^2^ | Studies/Patients | Any Grade | I^2^ | Studies/Patients | Severe Grade | I^2^ |
| PD-1 | 12/2589 | 0.0206 [0.0156; 0.0272] | 0.00% | 11/2522 | 0.0143 [0.0101; 0.0201] | 0.00% | 8/1195 | 0.1309 [0.0878; 0.1907] | 82.20% | 7/1128 | 0.0163 [0.0099; 0.0266] | 0.00% |
| Nivolumab | 5/955 | 0.0145 [0.0085; 0.0245] | 0.00% | 5/955 | 0.0090 [0.0046; 0.0176] | 0.00% | 5/955 | 0.1810 [0.1323; 0.2427] | 77.10% | 5/955 | 0.0175 [0.0105; 0.0290] | 0.00% |
| 3 mg/kg | 4/848 | 0.0130 [0.0072; 0.0234] | 0.00% | 4/848 | 0.0083 [0.0039; 0.0172] | 0.00% | 4/848 | 0.1826 [0.1240; 0.2607] | 82.80% | 4/848 | 0.0155 [0.0082; 0.0291] | 10.20% |
| Pembrolizumab | 7/1634 | 0.0211 [0.0145; 0.0307] | 11.60% | 6/1567 | 0.0167 [0.0110; 0.0253] | 4.20% | 3/240 | 0.0397 [0.0168; 0.0908] | 37.20% | 2/173 | 0.0057 [0.0008; 0.0394] | 0.00% |
| 2 mg/kg | 1/178 |  |  | 1/178 |  |  | 1/89 |  |  | 1/89 |  |  |
| 10 mg/kg | 2/456 | 0.0285 [0.0142; 0.0565] | 28.60% | 2/456 | 0.0208 [0.0105; 0.0408] | 5.40% | 1/84 |  |  | 1/84 |  |  |

**Table S17** Particular incidence of gastrointestinal irAEs in melanomas patients (continued)

| Target and Drug | Pancreatitis | | | | | |
| --- | --- | --- | --- | --- | --- | --- |
|  | Studies/Patients | Any Grade | I^2^ | Studies/Patients | Severe Grade | I^2^ |
| PD-1 | 4/828 | 0.0067 [0.0025; 0.0177] | 0.00% | 4/828 | 0.0067 [0.0025; 0.0177] | 0.00% |
| Nivolumab |  |  |  |  |  |  |
| 3 mg/kg |  |  |  |  |  |  |
| Pembrolizumab | 4/828 | 0.0067 [0.0025; 0.0177] | 0.00% | 4/828 | 0.0067 [0.0025; 0.0177] | 0.00% |
| 2 mg/kg | 1/89 |  |  | 1/89 |  |  |
| 10 mg/kg | 1/84 |  |  | 1/84 |  |  |

**Table S18** Particular incidence of hepatic irAEs in melanomas patients

| Target and Drug | AST increased | | | | | | ALT increased | | | | | |
| --- | --- | --- | --- | --- | --- | --- | --- | --- | --- | --- | --- | --- |
|  | Studies/Patients | Any Grade | I^2^ | Studies/Patients | Severe Grade | I^2^ | Studies/Patients | Any Grade | I^2^ | Studies/Patients | Severe Grade | I^2^ |
| PD-1 | 7/1128 | 0.0334 [0.0231; 0.0481] | 10.10% | 7/1128 | 0.0334 [0.0231; 0.0481] | 10.10% | 7/1128 | 0.0345 [0.0229; 0.0517] | 27.50% | 7/1128 | 0.0108 [0.0060; 0.0191] | 0.00% |
| Nivolumab | 5/955 | 0.0366 [0.0259; 0.0517] | 2.10% | 5/955 | 0.0366 [0.0259; 0.0517] | 2.10% | 5/955 | 0.0351 [0.0233; 0.0525] | 22.60% | 5/955 | 0.0114 [0.0063; 0.0208] | 0.00% |
| 3 mg/kg | 4/848 | 0.0355 [0.0224; 0.0557] | 26.50% | 4/848 | 0.0355 [0.0224; 0.0557] | 26.50% | 4/848 | 0.0328 [0.0199; 0.0537] | 35.90% | 4/848 | 0.0120 [0.0065; 0.0221] | 0.00% |
| Pembrolizumab | 2/173 | 0.0116 [0.0029; 0.0450] | 0.00% | 2/173 | 0.0116 [0.0029; 0.0450] | 0.00% | 2/173 | 0.0128 [0.0032; 0.0499] | 0.00% | 2/173 | 0.0057 [0.0008; 0.0394] | 0.00% |
| 2 mg/kg | 1/89 |  |  | 1/89 |  |  | 1/89 |  |  | 1/89 |  |  |
| 10 mg/kg | 1/84 |  |  | 1/84 |  |  | 1/84 |  |  | 1/84 |  |  |

**Table S18** Particular incidence of hepatic irAEs in melanomas patients (continued)

| Target and Drug | Hepatitis | | | | | |
| --- | --- | --- | --- | --- | --- | --- |
|  | Studies/Patients | Any Grade | I^2^ | Studies/Patients | Severe Grade | I^2^ |
| PD-1 | 8/1740 | 0.0129 [0.0084; 0.0197] | 0.0% | 8/1740 | 0.0114 [0.0071; 0.0182] | 0.0% |
| Nivolumab |  |  |  |  |  |  |
| 3 mg/kg |  |  |  |  |  |  |
| Pembrolizumab | 8/1740 | 0.0129 [0.0084; 0.0197] | 0.0% | 8/1740 | 0.0114 [0.0071; 0.0182] | 0.0% |
| 2 mg/kg | 2/267 | 0.0112 [0.0036; 0.0342] | 0.00% | 2/267 | 0.0076 [0.0019; 0.0297] | 0.00% |
| 10 mg/kg | 3/540 | 0.0166 [0.0085; 0.0322] | 0.00% | 3/540 | 0.0166 [0.0085; 0.0322] | 0.00% |

**Table S19** Particular incidence of pulmonary irAEs in melanomas patients

| Target and Drug | Pneumonitis | | | | | |
| --- | --- | --- | --- | --- | --- | --- |
|  | Studies/Patients | Any Grade | I^2^ | Studies/Patients | Severe Grade | I^2^ |
| PD-1 | 13/2695 | 0.0218 [0.0167; 0.0284] | 0.00% | 13/2695 | 0.0057 [0.0033; 0.0099] | 0.00% |
| Nivolumab | 5/955 | 0.0177 [0.0109; 0.0284] | 0.00% | 5/955 | 0.0041 [0.0014; 0.0115] | 0.00% |
| 3 mg/kg | 4/848 | 0.0168 [0.0100; 0.0282] | 0.00% | 4/848 | 0.0040 [0.0013; 0.0122] | 0.00% |
| Pembrolizumab | 8/1740 | 0.0202 [0.0133; 0.0304] | 21.40% | 8/1740 | 0.0065 [0.0034; 0.0124] | 0.00% |
| 2 mg/kg | 2/267 | 0.0152 [0.0057; 0.0399] | 0.00% | 2/267 | 0.0039 [0.0006; 0.0274] | 0.00% |
| 10 mg/kg | 3/540 | 0.0168 [0.0088; 0.0320] | 0.00% | 3/540 | 0.0092 [0.0037; 0.0230] | 0.00% |

**Table S20** Particular incidence of renal irAEs in melanomas patients

| Target and Drug | Blood creatinine increased | | | | | | Nephritis | | | | | |
| --- | --- | --- | --- | --- | --- | --- | --- | --- | --- | --- | --- | --- |
|  | Studies/Patients | Any Grade | I^2^ | Studies/Patients | Severe Grade | I^2^ | Studies/Patients | Any Grade | I^2^ | Studies/Patients | Severe Grade | I^2^ |
| PD-1 | 3/535 | 0.0138 [0.0031; 0.0600] | 68.70% | 3/535 | 0.0033 [0.0007; 0.0162] | 0.00% | 4/912 | 0.0063 [0.0026; 0.0150] | 0.00% | 4/912 | 0.0022 [0.0006; 0.0089] | 0.00% |
| Nivolumab | 3/535 | 0.0138 [0.0031; 0.0600] | 68.70% | 3/535 | 0.0033 [0.0007; 0.0162] | 0.00% |  |  |  |  |  |  |
| Pembrolizumab | |  |  |  |  |  | 4/912 | 0.0063 [0.0026; 0.0150] | 0.00% | 4/912 | 0.0022 [0.0006; 0.0089] | 0.00% |
| 2 mg/kg | |  |  |  |  |  | 1/178 |  |  | 1/178 |  |  |
| 10 mg/kg |  |  |  |  |  |  | 2/456 | 0.0045 [0.0011; 0.0178] | 0.00% | 2/456 | 0.0022 [0.0003; 0.0157] | 0.00% |

**Table S20** Particular incidence of renal irAEs in melanomas patients (continued)

| Target and Drug | Renal failure | | | | | |
| --- | --- | --- | --- | --- | --- | --- |
|  | Studies/Patients | Any Grade | I^2^ | Studies/Patients | Severe Grade | I^2^ |
| PD-1 | 2/313 | 0.0135 [0.0051; 0.0353] | 0.00% | 2/313 | 0.0135 [0.0051; 0.0353] | 0.00% |
| Nivolumab | 2/313 | 0.0135 [0.0051; 0.0353] | 0.00% | 2/313 | 0.0135 [0.0051; 0.0353] | 0.00% |
| Pembrolizumab |  |  |  |  |  |  |

1. **Incidence of irAEs in NSCLC patients**

**Table S21** Particular incidence of cutaneous irAEs with nivolumab (3 mg/kg), any grade and severe grade in NSCLC patients

|  | Studies/Patients | Any Grade | I^2^ | Studies/Patients | Severe Grade | I^2^ |
| --- | --- | --- | --- | --- | --- | --- |
| Pruritus | 4/587 | 0.0654 [0.0377; 0.1111] | 56.80% | 4/587 | 0.0071 [0.0023; 0.0217] | 0.00% |
| Rash | 4/587 | 0.0952 [0.0564; 0.1563] | 69.80% | 4/587 | 0.0131 [0.0040; 0.0424] | 50.00% |
| Rash maculopapular | 3/535 | 0.0156 [0.0078; 0.0309] | 0.00% | 3/535 | 0.003 [0.0006; 0.0149] | 0.00% |

**Table S22** Particular incidence of endocrinologic irAEs any grade and severe grade in NSCLC patients

| Target and Drug | Hypothyroidism | | | | | | Hyperthyroidism | | | | |  |
| --- | --- | --- | --- | --- | --- | --- | --- | --- | --- | --- | --- | --- |
|  | Studies/Patients | Any Grade | I^2^ | Studies/Patients | Severe Grade | I^2^ | Studies/Patients | Any Grade | I^2^ | Studies/Patients | Severe Grade | I^2^ |
| PD-1 | 10/2080 | 0.0746 [0.0595; 0.0931] | 43.90% | 9/1585 | 0.0035 [0.0014; 0.0087] | 0.00% | 5/1175 | 0.0409 [0.0237; 0.0698] | 67.10% | 5/1175 | 0.0035 [0.0012; 0.0099] | 0.00% |
| Nivolumab | 4/587 | 0.0528 [0.0360; 0.0768] | 7.30% | 4/587 | 0.0040 [0.0010; 0.0159] | 0.00% | 2/339 | 0.0149 [0.0062; 0.0352] | 0.00% | 2/339 | 0.0040 [0.0006; 0.0281] | 0.00% |
| 3 mg/kg | 4/587 | 0.0528 [0.0360; 0.0768] | 7.30% | 4/587 | 0.0040 [0.0010; 0.0159 | 0.00% | 2/339 | 0.0149 [0.0062; 0.0352] | 0.00% | 2/339 | 0.0040 [0.0006; 0.0281] | 0.00% |
| Pembrolizumab | 6/1493 | 0.0852 [0.0680; 0.1063] | 36.30% | 5/998 | 0.0031 [0.0009; 0.0105] | 0.00% | 3/836 | 0.0547 [0.0359; 0.0825] | 50.90% | 3/836 | 0.0033 [0.0010; 0.0113] | 0.00% |
| 2 mg/kg | 2/400 | 0.0785 [0.0557; 0.1095] | 0.00% | 2/400 | 0.0034 [0.0005; 0.0240] | 0.00% | 1/339 |  |  | 1/339 |  |  |
| 10 mg/kg | 1/343 |  |  | 1/343 |  |  | 1/343 |  |  | 1/343 |  |  |
| 200mg | 1/154 |  |  | 1/154 |  |  | 1/154 |  |  | 1/154 |  |  |

**Table S22** Particular incidence of endocrinologic irAEs any grade and severe grade in NSCLC patients (continued)

| Target and Drug | Hypophysitis | | | | | | Thyroiditis | | | | |  |
| --- | --- | --- | --- | --- | --- | --- | --- | --- | --- | --- | --- | --- |
|  | Studies/Patients | Any Grade | I^2^ | Studies/Patients | Severe Grade | I^2^ | Studies/Patients | Any Grade | I^2^ | Studies/Patients | Severe Grade | I^2^ |
| PD-1 | 4/937 | 0.0048 [0.0018; 0.0128] | 0.00% | 4/937 | 0.0048 [0.0018; 0.0128] | 0.00% | 6/1292 | 0.0133 [0.0056; 0.0311] | 54.60% | 6/1292 | 0.0028 [0.0009; 0.0086] | 0.00% |
| Nivolumab |  |  |  |  |  |  | 3/456 | 0.012 [0.0027; 0.0512] | 53.10% | 3/456 | 0.0041 [0.0008; 0.0201] | 0.00% |
| 3 mg/kg |  |  |  |  |  |  | 3/456 | 0.0120 [0.0027; 0.0512] | 53.10% | 3/456 | 0.0041 [0.0008; 0.0201] | 0.00% |
| Pembrolizumab | 4/937 | 0.0048 [0.0018; 0.0128] | 0.00% | 4/937 | 0.0048 [0.0018; 0.0128] | 0.00% | 3/836 | 0.010 [0.0024; 0.0416] | 66.80% | 3/836 | 0.0019 [0.0004; 0.0094] | 0.00% |
| 2 mg/kg | 1/339 |  |  | 1/339 |  |  | 1/339 |  |  | 1/339 |  |  |
| 10 mg/kg | 1/343 |  |  | 1/343 |  |  | 1/343 |  |  | 1/343 |  |  |
| 200mg | 1/154 |  |  | 1/154 |  |  | 1/154 |  |  | 1/154 |  |  |

**Table S22** Particular incidence of endocrinologic irAEs any grade and severe grade in NSCLC patients (continued)

| Target and Drug |  | Adrenal insufficiency | | | |  |
| --- | --- | --- | --- | --- | --- | --- |
|  | Studies/Patients | Any Grade | I^2^ | Studies/Patients | Severe Grade | I^2^ |
| PD-1 | 4/851 | 0.0087 [0.0042; 0.0182] | 0.00% | 4/851 | 0.0063 [0.0023; 0.0165] | 0.00% |
| Nivolumab | 2/169 | 0.0128 [0.0032; 0.0498] | 0.00% | 2/169 | 0.0118 [0.0030; 0.0459] | 0.00% |
| 3 mg/kg | 2/169 | 0.0128 [0.0032; 0.0498] | 0.00% | 2/169 | 0.0118 [0.0030; 0.0459] | 0.00% |
| Pembrolizumab | 2/682 | 0.0075 [0.0031; 0.0178] | 0.00% | 2/682 | 0.0033 [0.0008; 0.0132] | 0.00% |
| 2 mg/kg | 1/339 |  |  | 1/339 |  |  |
| 10 mg/kg | 1/343 |  |  | 1/343 |  |  |
| 200mg |  |  |  |  |  |  |

**Table S23** Particular incidence of gastrointestinal irAEs any grade and severe grade in NSCLC patients

| Target and Drug | Colitis | | | | | | Diarrhea | | | | | |
| --- | --- | --- | --- | --- | --- | --- | --- | --- | --- | --- | --- | --- |
|  | Studies/Patients | Any Grade | I^2^ | Studies/Patients | Severe Grade | I^2^ | Studies/Patients | Any Grade | I^2^ | Studies/Patients | Severe Grade | I^2^ |
| PD-1/PD-L1 | 9/2167 | 0.0115 [0.0067; 0.0199] | 33.60% | 9/2167 | 0.0109 [0.0063; 0.0190] | 22.20% |  |  |  |  |  |  |
| PD-1 | 7/1416 | 0.0134 [0.0075; 0.0241] | 27.30% | 7/1416 | 0.0099 [0.0055; 0.0179] | 0.00% | 4/587 | 0.0824 [0.0626; 0.1076] | 0.00% | 4/587 | 0.0824 [0.0626; 0.1076] | 0.00% |
| Nivolumab | 2/418 | 0.0072 [0.0023; 0.0220] | 0.00% | 2/418 | 0.0052 [0.0013; 0.0204] | 0.00% | 4/587 | 0.0824 [0.0626; 0.1076] | 0.00% | 4/587 | 0.0824 [0.0626; 0.1076] | 0.00% |
| 3 mg/kg | 2/418 | 0.0072 [0.0023; 0.0220] | 0.00% | 2/418 | 0.0052 [0.0013; 0.0204] | 0.00% | 4/587 | 0.0824 [0.0626; 0.1076] | 0.00% | 4/587 | 0.0824 [0.0626; 0.1076] | 0.00% |
| Pembrolizumab | 5/998 | 0.0158 [0.0077; 0.0321] | 39.50% | 5/998 | 0.0115 [0.0058; 0.0227] | 8.60% |  |  |  |  |  |  |
| 2 mg/kg | 2/400 | 0.0235 [0.0057; 0.0917] | 71.90% | 2/400 | 0.0160 [0.0044; 0.0563] | 52.10% |  |  |  |  |  |  |
| 10 mg/kg | 1/343 |  |  | 1/343 |  |  |  |  |  |  |  |  |
| 200mg | 1/343 |  |  | 1/343 |  |  |  |  |  |  |  |  |
| PD-L1 | 2/751 | 0.0068 [0.0016; 0.0280] | 53.10% | 2/751 | 0.0038 [0.0003; 0.0426] | 59.10% |  |  |  |  |  |  |
| Atezolizumab | 2/751 | 0.0068 [0.0016; 0.0280] | 53.10% | 2/751 | 0.0038 [0.0003; 0.0426] | 59.10% |  |  |  |  |  |  |
| 1200mg | 2/751 |  |  | 2/751 |  |  |  |  |  |  |  |  |

**Table S23** Particular incidence of gastrointestinal irAEs any grade and severe grade in NSCLC patients (continued)

| Target and Drug | Pancreatitis | | | | | |
| --- | --- | --- | --- | --- | --- | --- |
|  | Studies/Patients | Any Grade | I_2_ | Studies/Patients | Severe Grade | I^2^ |
| PD-1/PD-L1 |  |  |  |  |  |  |
| PD-1 |  |  |  |  |  |  |
| Nivolumab |  |  |  |  |  |  |
| Pembrolizumab | 3/836 | 0.0085 [0.0037; 0.0194] | 0.00% | 3/836 | 0.0067 [0.0027; 0.0168] | 0.00% |
| 2 mg/kg | 1/339 |  |  | 1/339 |  |  |
| 10 mg/kg | 1/343 |  |  | 1/343 |  |  |
| 200mg | 1/154 |  |  | 1/154 |  |  |
| PD-L1 |  |  |  |  |  |  |
| Atezolizumab |  |  |  |  |  |  |

**Table S24** Particular incidence of hepatic irAEs any grade and severe grade in NSCLC patients

| Target and Drug | AST increased | | | | | | ALT increased | | | | | |
| --- | --- | --- | --- | --- | --- | --- | --- | --- | --- | --- | --- | --- |
|  | Studies/Patients | Any Grade | I^2^ | Studies/Patients | Severe Grade | I^2^ | Studies/Patients | Any Grade | I^2^ | Studies/Patients | Severe Grade | I^2^ |
| PD-1／PD-L1 | 4/612 | 0.0311 [0.0197; 0.0488] | 0.00% | 4/612 | 0.0145 [0.0058; 0.0358] | 26.90% | 5/729 | 0.029 [0.0186; 0.0451] | 0.00% | 5/729 | 0.0125 [0.0045; 0.0340] | 28.20% |
| PD-1 | 3/470 | 0.0267 [0.0152; 0.0464] | 0.00% | 3/470 | 0.0098 [0.0028; 0.0344] | 27.30% | 4/587 | 0.0244 [0.0142; 0.0416] | 0.00% | 4/587 | 0.0077 [0.0019; 0.0296] | 25.10% |
| Nivolumab | 3/470 | 0.0267 [0.0152; 0.0464] | 0.00% | 3/470 | 0.0098 [0.0028; 0.0344] | 27.30% |  |  |  |  |  |  |
| 3 mg/kg | 3/470 | 0.0267 [0.0152; 0.0464] | 0.00% | 3/470 | 0.0098 [0.0028; 0.0344] | 27.30% |  |  |  |  |  |  |
| PD-L1 | 1/142 |  |  | 1/142 |  |  | 1/142 |  |  | 1/142 |  |  |
| Atezolizumab | 1/142 |  |  | 1/142 |  |  | 1/142 |  |  | 1/142 |  |  |
| 1200mg | 1/142 |  |  | 1/142 |  |  | 1/142 |  |  | 1/142 |  |  |

**Table S24** Particular incidence of hepatic irAEs any grade and severe grade in NSCLC patients (continued)

| Target and Drug |  | Blood bilirubin increased | | | | | | Hepatitis | | | | | |
| --- | --- | --- | --- | --- | --- | --- | --- | --- | --- | --- | --- | --- | --- |
|  | Studies/Patients | Any Grade | I^2^ | Studies/Patients | Severe Grade | I^2^ | Studies/Patients | | Any Grade | I^2^ | Studies/Patients | Severe Grade | I^2^ |
| PD-1／PD-L1 |  |  |  |  |  |  |  | |  |  |  |  |  |
| PD-1 |  |  |  |  |  |  |  | |  |  |  |  |  |
| Nivolumab | 2/418 | 0.0048 [0.0012; 0.0190] | 0.00% | 2/418 | 0.0026 [0.0004; 0.0180] | 0.00% |  | |  |  |  |  |  |
| 3 mg/kg | 2/418 | 0.0048 [0.0012; 0.0190] | 0.00% | 2/418 | 0.0026 [0.0004; 0.0180] | 0.00% |  | |  |  |  |  |  |
| PD-L1 |  |  |  |  |  |  | 2/751 | | 0.0042 [0.0014; 0.0130] | 0.00% | 2/751 | 0.004 [0.0013; 0.0123] | 0.00% |
| Atezolizumab |  |  |  |  |  |  | 2/751 | | 0.0042 [0.0014; 0.0130] | 0.00% | 2/751 | 0.004 [0.0013; 0.0123] | 0.00% |
| 1200mg |  |  |  |  |  |  |  | |  |  |  |  |  |

**Table S25** Particular incidence of pulmonary irAEs any grade and severe grade in NSCLC patients

| Target and Drug | Pneumonitis | | | | | |
| --- | --- | --- | --- | --- | --- | --- |
|  | Studies/Patients | Any Grade | I^2^ | Studies/Patients | Severe Grade | I^2^ |
| PD-1／PD-L1 | 12/2831 | 0.0359 [0.0273; 0.0471] | 39.00% | 12/2831 | 0.017 [0.0126; 0.0229] | 0.00% |
| PD-1 | 10/2080 | 0.0415 [0.0336; 0.0512] | 0.00% | 10/2080 | 0.0193 [0.0141; 0.0264] | 0.00% |
| Nivolumab | 4/587 | 0.0388 [0.0257; 0.0582] | 0.00% | 4/587 | 0.0152 [0.0076; 0.0300] | 0.00% |
| 3 mg/kg | 4/587 | 0.0388 [0.0257; 0.0582] | 0.00% | 4/587 | 0.0152 [0.0076; 0.0300] | 0.00% |
| Pembrolizumab | 6/1493 | 0.0426 [0.0333; 0.0544] | 0.00% | 6/1493 | 0.0206 [0.0144; 0.0293] | 0.00% |
| 2 mg/kg | 2/400 | 0.0453 [0.0287; 0.0708] | 0.00% | 2/400 | 0.0229 [0.0119; 0.0434] | 0.00% |
| 10 mg/kg | 1/343 |  |  | 1/343 |  |  |
| 200mg | 1/154 |  |  | 1/154 |  |  |
| PD-L1 | 2/751 | 0.0160 [0.0057; 0.0443] | 62.80% | 2/751 | 0.0067 [0.0028; 0.0159] | 0.00% |
| Atezolizumab | 2/751 | 0.0160 [0.0057; 0.0443] | 62.80% | 2/751 | 0.0067 [0.0028; 0.0159] | 0.00% |
| 1200mg | 2/751 |  |  | 2/751 |  |  |

**Table S26** Particular incidence of renal irAEs with nivolumab (3 mg/kg), any grade and severe grade in NSCLC patients

|  | Studies/Patients | Any Grade | I^2^ | Studies/Patients | Severe Grade | I^2^ |
| --- | --- | --- | --- | --- | --- | --- |
| Blood creatinine increased | 3/535 | 0.0213 [0.0118; 0.0380] | 0.00% | 3/535 | 0.0213 [0.0118; 0.0380] | 0.00% |
| Renal failure acute | 2/285 | 0.0055 [0.0014; 0.0215] | 0.00% | 2/285 | 0.0027 [0.0004; 0.0190] | 0.00% |
| Renal failure | 2/404 | 0.0055 [0.0014; 0.0215] | 0.00% | 2/404 | 0.0027 [0.0004; 0.0190] | 0.00% |

1. **Incidence of irAEs in RCC patients**

**Table S27** Particular incidence of cutaneous irAEs with nivolumab, any grade and severe grade in RCC patients

| Nivolumab | Pruritus | | | | | | Rash | | | | | |
| --- | --- | --- | --- | --- | --- | --- | --- | --- | --- | --- | --- | --- |
|  | Studies/Patients | Any Grade | I^2^ | Studies/Patients | Severe Grade | I^2^ | Studies/Patients | Any Grade | I^2^ | Studies/Patients | Severe Grade | I^2^ |
| All dosage | 8/293 | 0.1337 [0.0987; 0.1785] | 0.00% | 8/293 | 0.0231 [0.0104; 0.0504] | 0.00% | 8/293 | 0.1291 [0.0864; 0.1886] | 33.50% | 8/293 | 0.0144 [0.0054; 0.0378] | 0.00% |
| 0.3 mg/kg | 2/82 | 0.1107 [0.0586; 0.1994] | 0.00% | 2/82 | 0.0133 [0.0019; 0.0887] | 0.00% | 2/82 | 0.1385 [0.0484; 0.3367] | 65.40% | 2/82 | 0.0133 [0.0019; 0.0887] | 0.00% |
| 2 mg/kg | 2/76 | 0.1248 [0.0628; 0.2328] | 13.20% | 2/76 | 0.0258 [0.0065; 0.0972] | 0.00% | 2/76 | 0.0793 [0.0360; 0.1655] | 0.00% | 2/76 | 0.0140 [0.0020; 0.0931] | 0.00% |
| 10 mg/kg | 1/54 |  |  | 1/54 |  |  | 1/54 |  |  | 1/54 |  |  |
| 10 mg/kg naïve | 1/24 |  |  | 1/24 |  |  | 1/24 |  |  | 1/24 |  |  |
| 10 mg/kg treated | 1/23 |  |  | 1/23 |  |  | 1/23 |  |  | 1/23 |  |  |

**Table S28** Particular incidence of endocrinologic irAEs with nivolumab, any grade and severe grade in RCC patients

| Nivolumab | Hypothyroidism | | | | | |
| --- | --- | --- | --- | --- | --- | --- |
|  | Studies/Patients | Any Grade | I^2^ | Studies/Patients | Severe Grade | I^2^ |
| All dosage | 8/293 | 0.0732 [0.0477; 0.1109] | 0.00% | 8/293 | 0.0183 [0.0076; 0.0432] | 0.00% |
| 0.3 mg/kg | 2/82 | 0.0369 [0.0120; 0.1084] | 0.00% | 2/82 | 0.0133 [0.0019; 0.0887] | 0.00% |
| 2 mg/kg | 2/76 | 0.0671 [0.0282; 0.1514] | 0.00% | 2/76 | 0.0258 [0.0065; 0.0972] | 0.00% |
| 10 mg/kg | 1/54 |  |  | 1/54 |  |  |
| 10 mg/kg naïve | 1/24 |  |  | 1/24 |  |  |
| 10 mg/kg treated | 1/23 |  |  | 1/23 |  |  |

**Table S29** Particular incidence of gastrointestinal irAEs with nivolumab, any grade and severe grade in RCC patients

| Nivolumab | Colitis | | | | | | Diarrhea | | | | | |
| --- | --- | --- | --- | --- | --- | --- | --- | --- | --- | --- | --- | --- |
|  | Studies/Patients | Any Grade | I^2^ | Studies/Patients | Severe Grade | I^2^ | Studies/Patients | Any Grade | I^2^ | Studies/Patients | Severe Grade | I^2^ |
| All dosage | 4/91 | 0.0782 [0.0354; 0.1641] | 0.00% | 4/91 | 0.0635 [0.0265; 0.1441] | 0.00% | 8/293 | 0.167 [0.1098; 0.2457] | 51.80% | 8/293 | 0.0278 [0.0125; 0.0607] | 0.00% |
| 0.3 mg/kg | 1/22 |  |  | 1/22 |  |  | 2/82 | 0.0848 [0.0148; 0.3644] | 76.30% | 2/82 | 0.0133 [0.0019; 0.0887] | 0.00% |
| 2 mg/kg | 1/22 |  |  | 1/22 |  |  | 2/76 | 0.1346 [0.0739; 0.2328] | 0.00% | 2/76 | 0.0140 [0.0020; 0.0931] | 0.00% |
| 10 mg/kg |  |  |  |  |  |  | 1/54 |  |  | 1/54 |  |  |
| 10 mg/kg naïve | 1/24 |  |  | 1/24 |  |  | 1/24 |  |  | 1/24 |  |  |
| 10 mg/kg treated | 1/23 |  |  | 1/23 |  |  | 1/23 |  |  | 1/23 |  |  |

**Table S30** Particular incidence of hepatic irAEs with nivolumab, any grade and severe grade in RCC patients

| Nivolumab | AST increased | | | | | | ALT increased | | | | | |
| --- | --- | --- | --- | --- | --- | --- | --- | --- | --- | --- | --- | --- |
|  | Studies/Patients | Any Grade | I^2^ | Studies/Patients | Severe Grade | I^2^ | Studies/Patients | Any Grade | I^2^ | Studies/Patients | Severe Grade | I^2^ |
| All dosage | 7/259 | 0.0296 [0.0128; 0.0667] | 0.00% | 7/259 | 0.0415 [0.0212; 0.0795] | 0.00% | 8/293 | 0.0778 [0.0512; 0.1166] | 0.00% | 8/293 | 0.0349 [0.0182; 0.0657] | 0.00% |
| 0.3 mg/kg | 2/82 | 0.0133 [0.0019; 0.0887] | 0.00% | 2/82 | 0.0274 [0.0068; 0.1033] | 0.00% | 2/82 | 0.0547 [0.0200; 0.1410] | 5.90% | 2/82 | 0.0274 [0.0068; 0.1033] | 0.00% |
| 2 mg/kg | 2/76 | 0.0140 [0.0020; 0.0931] | 0.00% | 2/76 | 0.0258 [0.0065; 0.0972] | 0.00% | 2/76 | 0.0401 [0.0130; 0.1173] | 0.00% | 2/76 | 0.0258 [0.0065; 0.0972] | 0.00% |
| 10 mg/kg | 1/54 |  |  | 1/54 |  |  | 1/54 |  |  | 1/54 |  |  |
| 10 mg/kg naïve | 1/24 |  |  | 1/24 |  |  | 1/24 |  |  | 1/24 |  |  |
| 10 mg/kg treated | 1/23 |  |  | 1/23 |  |  | 1/23 |  |  | 1/23 |  |  |

**Table S30** Particular incidence of hepatic irAEs with nivolumab, any grade and severe grade in RCC patients (continued)

| Nivolumab | Blood bilirubin increased | | | | | |
| --- | --- | --- | --- | --- | --- | --- |
|  | Studies/Patients | Any Grade | I^2^ | Studies/Patients | Severe Grade | I^2^ |
| All dosage | 4/91 | 0.0782 [0.0354; 0.1641] | 0.00% | 4/91 | 0.0361 [0.0117; 0.1064] | 0.00% |
| 0.3 mg/kg | 1/22 |  |  | 1/22 |  |  |
| 2 mg/kg | 1/22 |  |  | 1/22 |  |  |
| 10 mg/kg |  |  |  |  |  |  |
| 10 mg/kg naïve | 1/24 |  |  | 1/24 |  |  |
| 10 mg/kg treated | 1/23 |  |  | 1/23 |  |  |

**Table S31** Particular incidence of pulmonary irAEs with nivolumab, any grade and severe grade in RCC patients

| Nivolumab | Pneumonitis | | | | | | Lung infiltration | | | | | |
| --- | --- | --- | --- | --- | --- | --- | --- | --- | --- | --- | --- | --- |
|  | Studies/Patients | Any Grade | I^2^ | Studies/Patients | Severe Grade | I^2^ | Studies/Patients | Any Grade | I^2^ | Studies/Patients | Severe Grade | I^2^ |
| All dosage | 7/259 | 0.0719 [0.0454; 0.1121] | 0.00% | 7/259 | 0.03 [0.0130; 0.0677] | 0.00% | 7/259 | 0.074 [0.0367; 0.1434] | 45.20% | 7/259 | 0.0145 [0.0051; 0.0405] | 0.00% |
| 0.3 mg/kg | 2/82 | 0.0488 [0.0184; 0.1230] | 0.00% | 2/82 | 0.0316 [0.0044; 0.1945] | 40.00% | 2/82 | 0.0449 [0.0083; 0.2078] | 50.30% | 2/82 | 0.0133 [0.0019; 0.0887] | 0.00% |
| 2 mg/kg | 2/76 | 0.0401 [0.0130; 0.1173] | 0.00% | 2/76 | 0.0140 [0.0020; 0.0931] | 0.00% | 2/76 | 0.0580 [0.0026; 0.5945] | 78.60% | 2/76 | 0.0140 [0.0020; 0.0931] | 0.00% |
| 10 mg/kg | 1/54 |  |  | 1/54 |  |  | 1/54 |  |  | 1/54 |  |  |
| 10 mg/kg naïve | 1/24 |  |  | 1/24 |  |  | 1/24 |  |  | 1/24 |  |  |
| 10 mg/kg treated | 1/23 |  |  | 1/23 |  |  | 1/23 |  |  | 1/23 |  |  |

**Table S32** Particular incidence of renal irAEs with nivolumab, any grade and severe grade in RCC patients

| Nivolumab | Blood creatinine increased | | | | | | Renal failure acute | | | | | |
| --- | --- | --- | --- | --- | --- | --- | --- | --- | --- | --- | --- | --- |
|  | Studies/Patients | Any Grade | I^2^ | Studies/Patients | Severe Grade | I^2^ | Studies/Patients | Any Grade | I^2^ | Studies/Patients | Severe Grade | I^2^ |
| All dosage | 7/259 | 0.074 [0.0367; 0.1434] | 45.20% | 7/259 | 0.0145 [0.0051; 0.0405] | 0.00% | 4/91 | 0.0714 [0.0324; 0.1504] | 0.00% | 4/91 | 0.0481 [0.0181; 0.1214] | 0.00% |
| 0.3 mg/kg | 2/82 | 0.0449 [0.0083; 0.2078] | 50.30% | 2/82 | 0.0133 [0.0019; 0.0887] | 0.00% | 1/22 |  |  | 1/22 |  |  |
| 2 mg/kg | 2/76 | 0.0580 [0.0026; 0.5945] | 78.60% | 2/76 | 0.0140 [0.0020; 0.0931] | 0.00% | 1/22 |  |  | 1/22 |  |  |
| 10 mg/kg | 1/54 |  |  | 1/54 |  |  |  |  |  |  |  |  |
| 10 mg/kg naïve | 1/24 |  |  | 1/24 |  |  | 1/24 |  |  | 1/24 |  |  |
| 10 mg/kg treated | 1/23 |  |  | 1/23 |  |  | 1/23 |  |  | 1/23 |  |  |

1. **Incidence of irAEs in urothelial carcinomas patients**

**Table S33** Incidence of irAEs with pembrolizumab and atezolizumab, any grade and severe grade in urothelial carcinomas patients

| Drug | All | | | | | | Rash | | | | | |
| --- | --- | --- | --- | --- | --- | --- | --- | --- | --- | --- | --- | --- |
|  | Studies/Patients | Any Grade | I^2^ | Studies/Patients | Severe Grade | I^2^ | Studies/Patients | Any Grade | I^2^ | Studies/Patients | Severe Grade | I^2^ |
| Pembrolizumab | 2/299 | 0.1706 [0.1321; 0.2176] | 0.00% | 2/299 | 0.0686 [0.0255; 0.1716] | 67.60% |  |  |  |  |  |  |
| Atezolizumab | 2/429 | 0.0909 [0.0575; 0.1409] | 50.60% | 2/429 | 0.0542 [0.0363; 0.0803] | 0.00% | 2/429 | 0.0159 [0.0031; 0.0768] | 72.90% | 1/119 |  |  |

**Table S33** Incidence of irAEs with pembrolizumab and atezolizumab, any grade and severe grade in urothelial carcinomas patients (continued)

| Drug | Colitis | | | | | |
| --- | --- | --- | --- | --- | --- | --- |
|  | Studies/Patients | Any Grade | I^2^ | Studies/Patients | Severe Grade | I^2^ |
| Pembrolizumab | 2/299 | 0.0235 [0.0113; 0.0485] | 0.00% | 2/299 | 0.0144 [0.0054; 0.0378] | 0.00% |
| Atezolizumab | 1/119 |  |  | 1/119 |  |  |

**Table S33** Incidence of irAEs with pembrolizumab and atezolizumab, any grade and severe grade in urothelial carcinomas patients (continued)

| Drug | AST increased | | | | | | ALT increased | | | | | |
| --- | --- | --- | --- | --- | --- | --- | --- | --- | --- | --- | --- | --- |
|  | Studies/Patients | Any Grade | I^2^ | Studies/Patients | Severe Grade | I^2^ | Studies/Patients | Any Grade | I^2^ | Studies/Patients | Severe Grade | I^2^ |
| Pembrolizumab |  |  |  |  |  |  |  |  |  |  |  |  |
| Atezolizumab | 2/429 | 0.007 [0.0023; 0.0216] | 0.00% | 1/119 |  |  | 2/429 | 0.0104 [0.0039; 0.0274] | 0.00% | 1/119 |  |  |
