# Supplementary material for: Immune-Related Adverse Events Associated with Anti-PD-1/PD-L1 Treatment for Malignancies: A Meta-Analysis
Source: Front Pharmacol. 2017 Oct 18;8:730. doi: 10.3389/fphar.2017.00730 (PMC5651530; doi:10.3389/fphar.2017.00730)
Supplement: Supplementary file 2 [file Presentation1.pdf]

Supplementary Figure legends

Figure S1 Incidence of global irAEs with anti-PD-1/ anti-PD-L1, any grade (A) and severe grade(B)

Figure S2 Incidence of global irAEs with nivolumab and pembrolizumab all dosage, any grade (A) and severe grade (B) in melanomas patients

Figure S3 Risk ratio of developing a global irAE with pembrolizumab at 10mg/kg comparing with 2mg/kg for global irAEs any grade

Figure S4 Quality assessment-graph(A) and summery (B)

Figure S5 Publication bias-The funnel plot (A) and Egger's funnel plot (B)
