# Supplementary figures and images for: Immune-Related Adverse Events Associated with Anti-PD-1/PD-L1 Treatment for Malignancies: A Meta-Analysis
Source: Front Pharmacol. 2017 Oct 18;8:730. doi: 10.3389/fphar.2017.00730 (PMC5651530; doi:10.3389/fphar.2017.00730)

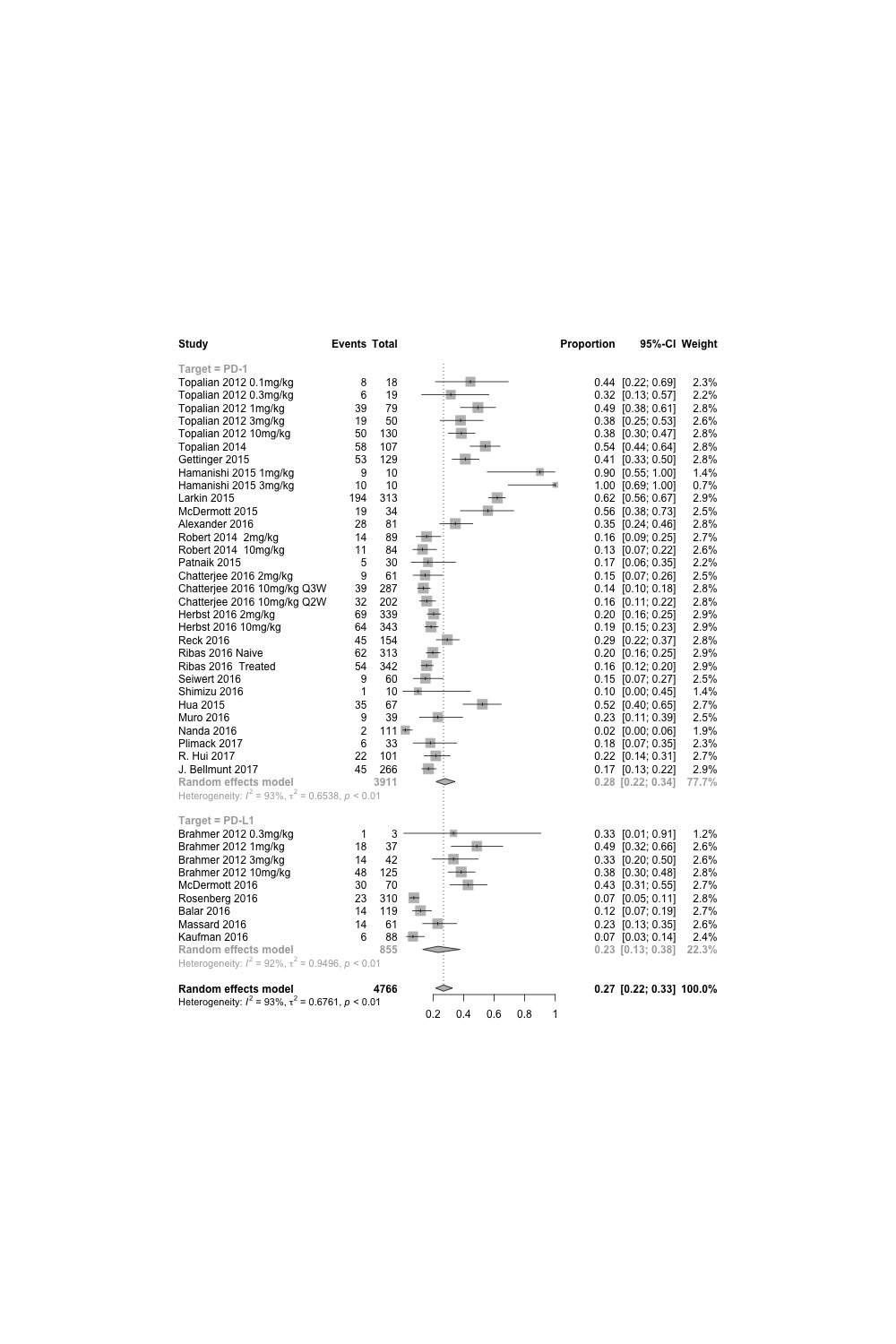

Supplement: Supplementary file 3 [file FigureS1A.JPEG]

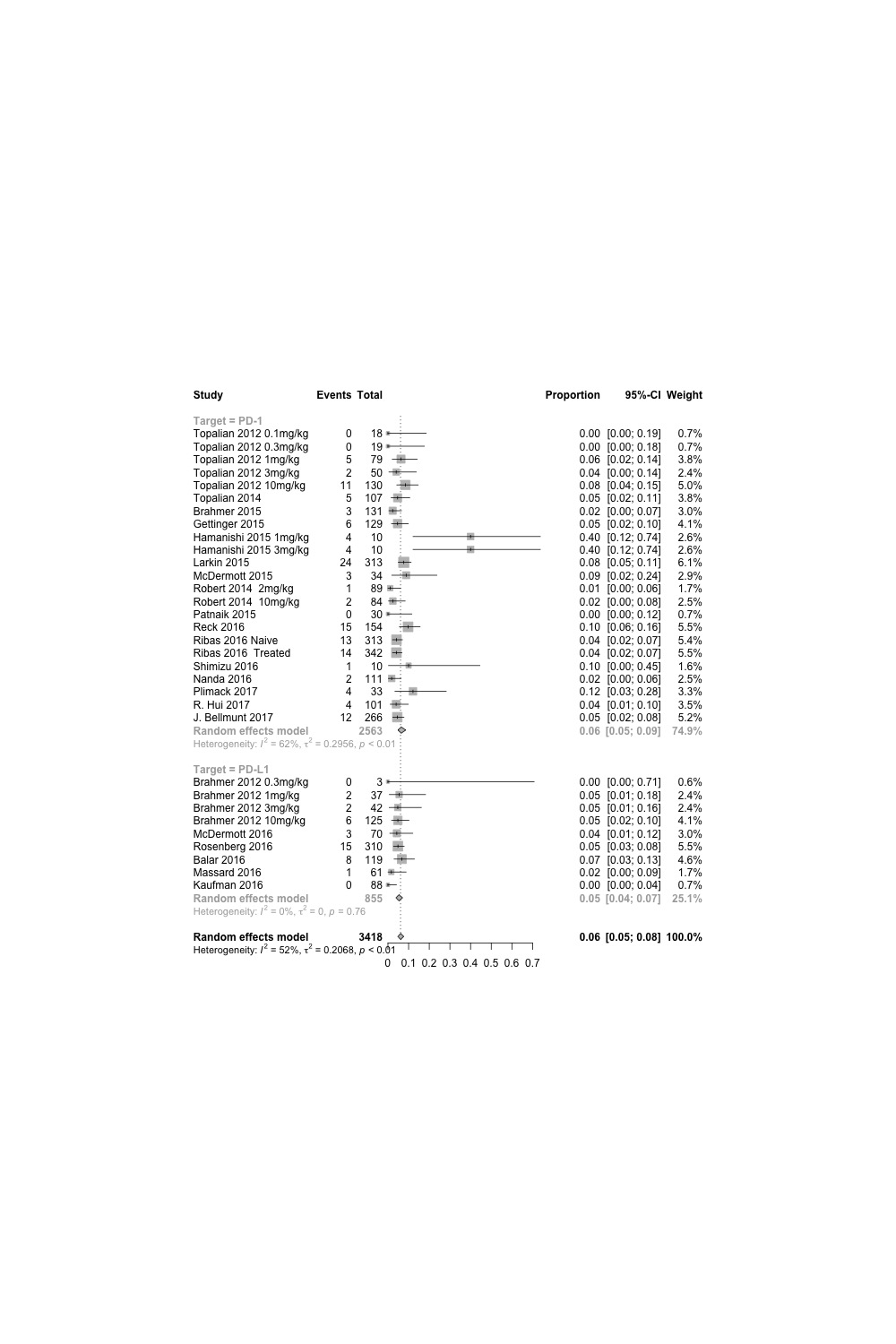

Supplement: Supplementary file 4 [file FigureS1B.JPEG]

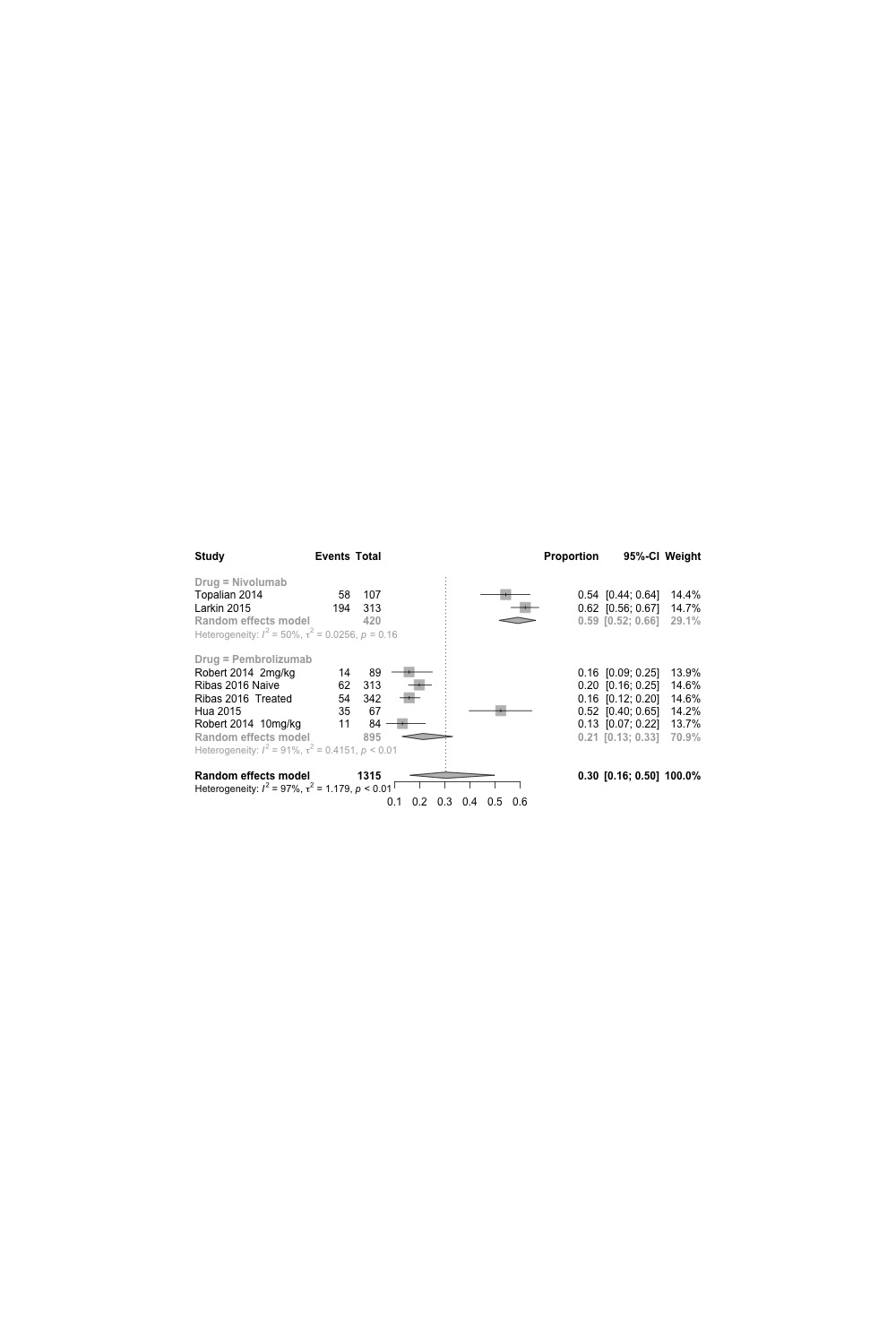

Supplement: Supplementary file 5 [file FigureS2A.JPEG]

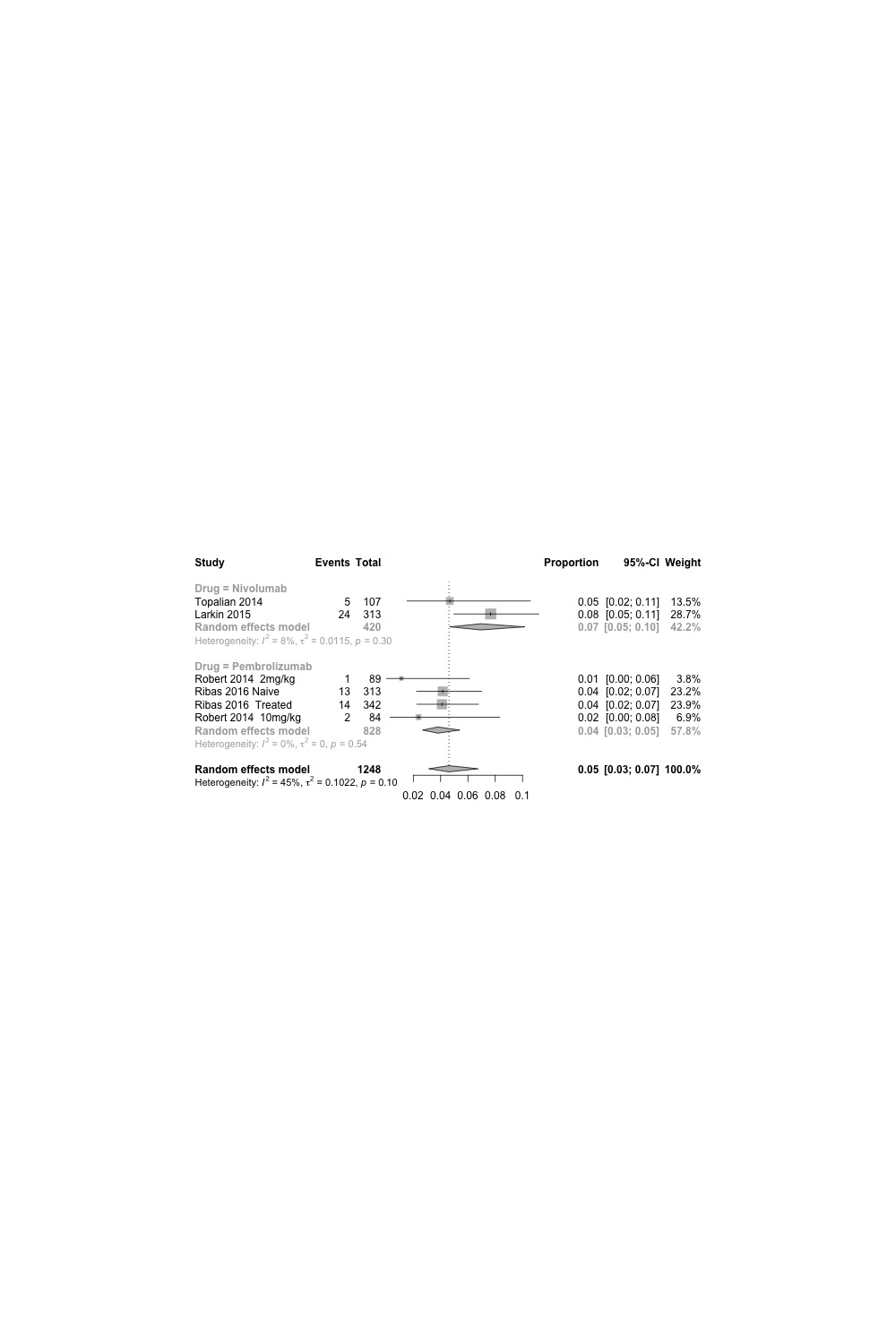

Supplement: Supplementary file 6 [file FigureS2B.JPEG]

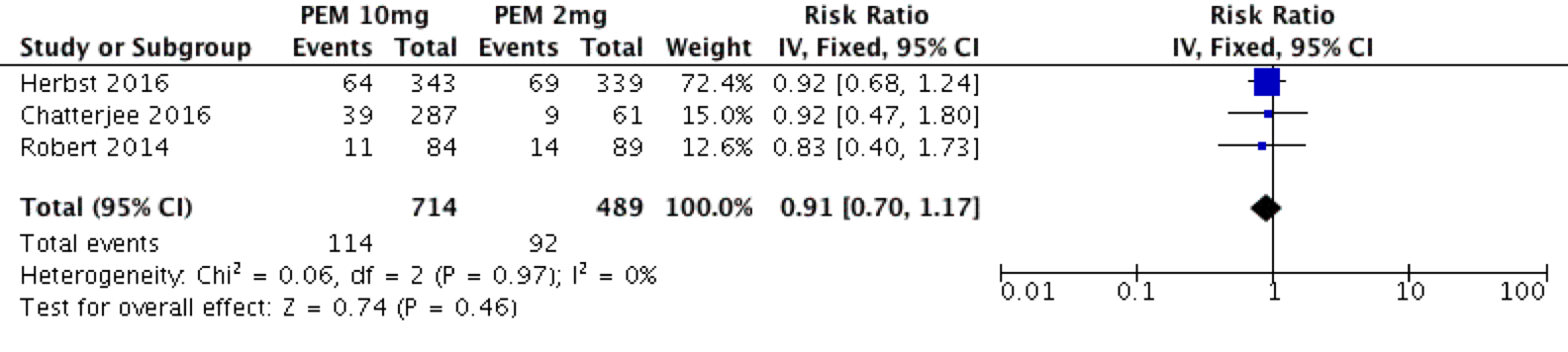

Supplement: Supplementary file 7 [file FigureS3.PNG]

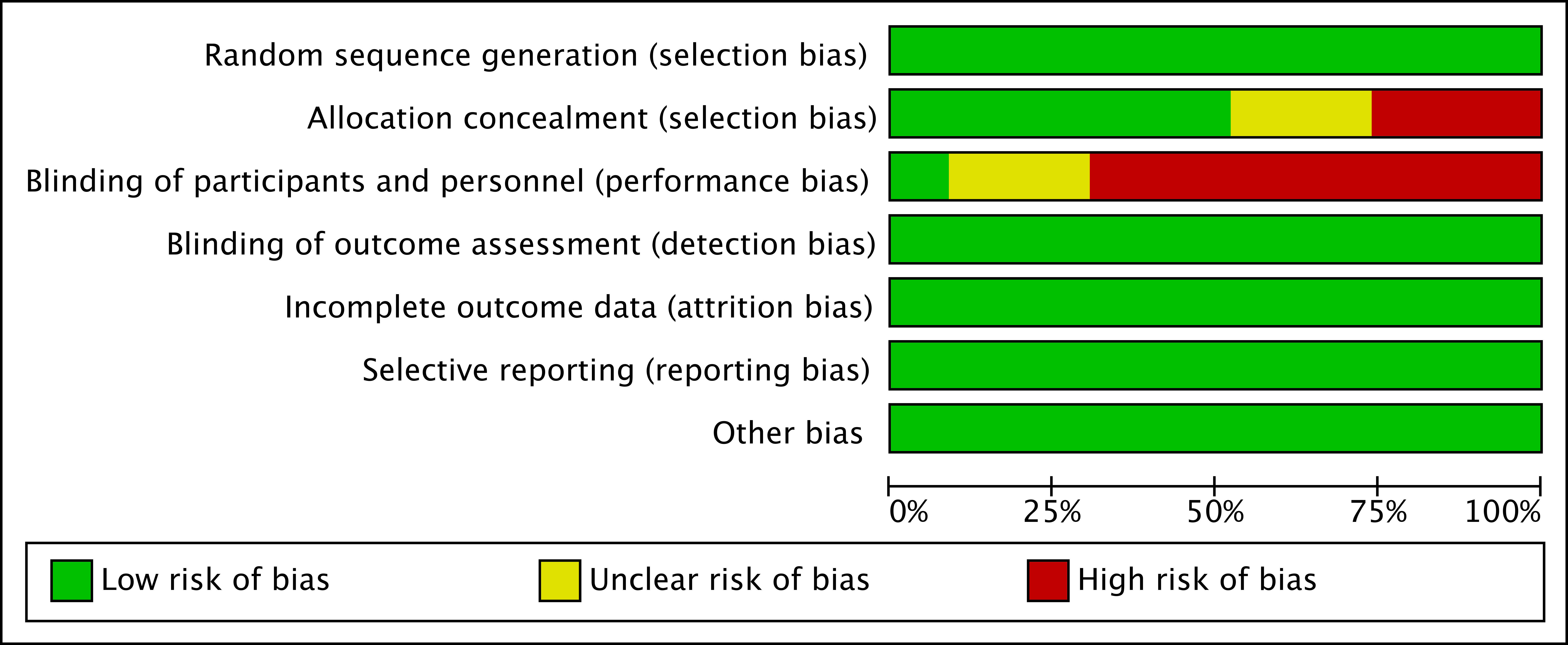

Supplement: Supplementary file 8 [file FigureS4A.JPEG]

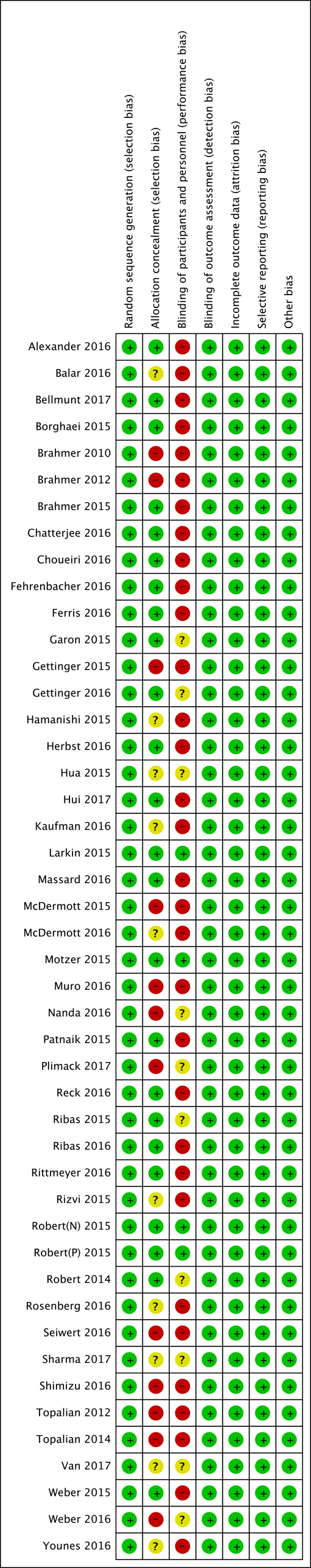

Supplement: Supplementary file 9 [file FigureS4B.JPEG]

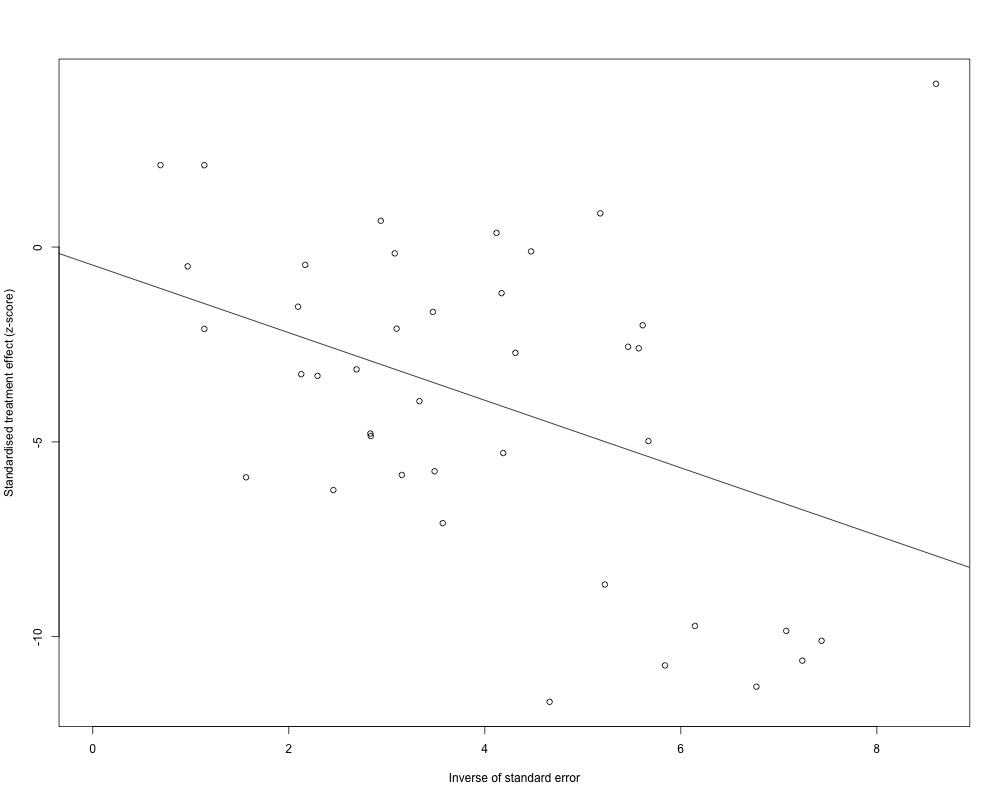

Supplement: Supplementary file 10 [file FigureS5A.JPEG]
